# Mutations in GTPBP3 cause aberrant mitochondrial respiration associated with combined oxidative phosphorylation deficiency 23

**DOI:** 10.1016/j.gendis.2024.101232

**Published:** 2024-02-02

**Authors:** Qianqian Li, Yang Yang, Ranran Li, Chenguang Yu, Kaidi Ren, Yin Feng, Xing Chen, Yi Luan, Xiangdong Kong

**Affiliations:** a Genetics and Prenatal Diagnosis Center, Department of Obstetrics and Gynecology, First Affiliated Hospital of Zhengzhou University, Zhengzhou, Henan 450000, China; b Clinical Systems Biology Laboratories, First Affiliated Hospital of Zhengzhou University, Zhengzhou, Henan 450000, China; c School of Life Science and Technology, Xinxiang Medical University, Xinxiang, Henan 453000, China; d Key Laboratory of Molecular Biophysics of the Ministry of Education, Cardio-X Center, College of Life Science and Technology and Center for Human Genome Research, Huazhong University of Science and Technology, Wuhan, Hubei 430000, China; e Department of Pharmacy, First Affiliated Hospital of Zhengzhou University, Zhengzhou, Henan 450000, China; f Department of Translational Medicine Center, First Affiliated Hospital of Zhengzhou University, Zhengzhou, Henan 450000, China

Combined oxidative phosphorylation deficiency 23 (COXPD23, MIM# 616198) is a rare autosomal-recessive mitochondrial disorder with variable disease severity ranging from death in early infancy to survival into the second decade of life,^1^ with the clinical symptoms of hypertrophic cardiomyopathy (HCM) and/or neurological symptoms with onset in early childhood, hypotonia, delayed psychomotor development, lactic acidosis,^1^ abnormal lesions in the basal ganglia, thalamus, and brainstem. COXPD23 is caused by homozygous or compound heterozygous mutations in the GTP-binding protein 3 (*GTPBP3*, OMIM∗ 608536) gene. Except for COXPD23, mutations in *GTPBP3* are also associated with other diseases^2^, ^3^, ^4^ (Table S1).

Herein, two compound heterozygous mutations in *GTPBP3* were each identified in two unrelated individuals with COXPD23. Three of the four mutations are novel. The four mutations disrupted the normal function of GTPBP3 in regulating cell respiration and mitochondrial protein expression at different levels.

This study was approved by the ethics committee of the First Affiliated Hospital of Zhengzhou University (Ethics No.: 2021-KY-0291-002). Written informed consent to participate was provided by the participants’ legal guardian/next of kin. Patient #1 was a boy of 6 months and 18 days with a poor spirit, hyperspasmia, increased serum lactate, liver failure, hypokalemia, myocardial injury, and moderate anemia. Patient #2 was a boy of 1 month and 6 days with severe pneumonia, (upper) cleft palate, congenital laryngeal chondromalacia, respiratory failure, and atrial septal defect (detailed presentation in supplementary information). The family trees are shown in Figure 1A and C. After filtering (Fig. S1), compound heterozygous mutations in *GTPBP3* (NM_133644.4) of c.785A > C(p.Q262P) and c.872A > T(p.N291I) (Figs. S2–7) were identified in patient #1, while compound heterozygous mutations of c.566G > A(p.R189H) and c.1528G > C(p.E510Q) (Fig. S8, 9) were identified in patient #2, verified via Sanger sequencing (Fig. 1B, D) with primer pairs in Table S3. The pathogenicity of the four mutations was shown in Table S4 according to American College of Medical Genetics and Genomics guidelines.

**Figure 1:**
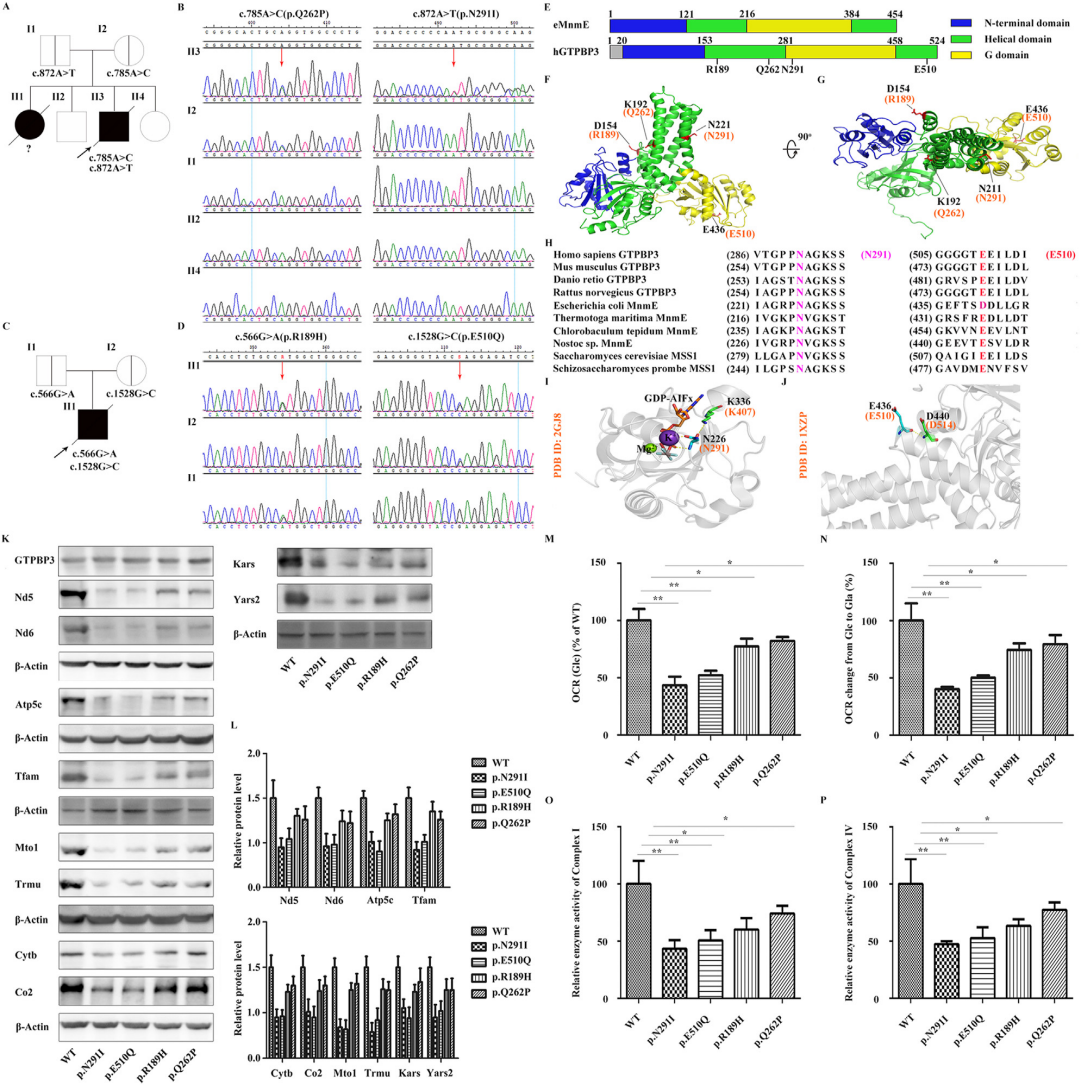
The four mutations impaired the normal function of GTPBP3 in regulating cell respiration and mitochondrial protein expression at different levels. **(A, C)** Family trees. **(B, D)** Sanger sequencing results of the two compound heterozygous mutations in *GTPBP3* (NM_133644.3) (red arrows). **(E)** The schematic diagram showing the domain compositions of hGTPBP3 and eMnmE based on the crystal structure of tMnmE (PDB: 1XZP). Pathogenic mutations were localized in the hGTPBP3 domains. The N-terminal domain (blue), helical domain (green), and G domain (yellow) are shown. **(F)** Pathogenic mutations were localized in the structure of tMnmE (PDB: 1XZP). The N-terminal domain (blue), helical domain (green), and G domain (yellow) were indicated. Black and orange indicate the amino acid residues of tMnmE and hGTPBP3, respectively. **(G)** Location of the pathogenic mutations in the structure of tMnmE (PDB: 1XZP) as viewed from the top. **(H)** Primary sequence alignment of GTPBP3/MnmE/MSS1 in different species. Black arrows indicate the amino acid residues N291 and E510 of hGTPBP3. h, *Homo sapiens*; m, *Mus musculus*; d, *Danio rerio*; r, *Rattus norvegicus*; e, *Escherichia coli*; t, *Thermotoga maritima*; c, *Chlorobaculum tepidum*; n, *Nostoc* sp.; sa, *Saccharomyces cerevisiae*; sc, *Schizosaccharomyces prombe*. **(I)** Three-dimensional model of the hGTPBP3 G domain based on the eMnmE G domain structure (PDB: 2GJ8). N291 (orange) is essential for GTPase activity and interacts with R407 (orange). GDP-AIFx (orange), K^+^ (purple), and Mg2^+^ (green) are displayed. Note that eMnmE counterparts (N226 and K336) are shown in black. **(J)** E510 in hGTPBP3 is conserved and interacts with D514 based on the structure of tMnmE (PDB: 1XZP). **(K, L)** The protein levels of OXPHOS in WT GTPBP3 and MU GTPBP3 cells, detected using Western blot assays: four mtDNA-encoding polypeptides (Nd5, Nd6, Cytb, and Co2), the nuclear-encoding protein Atp5c, two proteins involved in tRNA modification and protein synthesis (Mto1 and Trmu), and three mitochondrial proteins of non-OXPHOS subunits (Kars, Yars2, and Tfam). **(M)** The oxygen consumption rate (OCR) of lung epithelial cells expressing WT or MU *GTPBP3* cultured in a high-glucose (Glc) medium. OCR was expressed as a percentage relative to that of the WT. **(N)** The OCR of lung epithelial cells expressing WT or MU *GTPBP3* and cultured in a galactose (Gal) growth medium. **(O, P)** Activities of respiratory chain complexes I and IV in cells transfected with WT or MU *GTPBP3*. ∗*P* < 0.05, ∗∗*P* < 0.01.

MnmE (formerly TrmE) is a guanine nucleotide-binding protein conserved between bacteria and humans that binds to and hydrolyzes GTP. It contains a canonical G domain and is conserved in all three kingdoms of life. MnmE is the homolog of human *GTPBP3*. The location of key pathogenic mutations in the hGTPBP3 domains is shown in Figure 1E–G based on the crystal structure of tMnmE (Protein Data Bank, PDB: 1XZP). Among the four mutations, N291 and E510, which play important roles in hGTPBP3 activity, were evolutionarily conserved as observed through sequence alignment (Fig. 1H). In the structure of the eMnmE G domain (PDB: 2GJ8), the hexagonal coordination of potassium is complemented by the side-chain CO of N226 (hGTPBP3 N291 counterpart), the α- and β-phosphate oxygens of GDP (Fig. 1I). Meanwhile, N226 also interacts with K336 (Fig. 1I). Additionally, in the structure of tMnmE (PDB: 1XZP), E436 (hGTPBP3 N510 counterpart) has a polar interaction with D440 (Fig. 1J). As GTPBP3 is the human homolog of MnmE, and the residues N291 and E510 in hGTPBP3 are conserved in different species, we propose that N291 cooperatively interacts with potassium and K407 in the G domain and E510 interacts with D514 in hGTPBP3.

The most obvious symptoms of patients with mutated *GTPBP3* are associated with abnormal respiratory functions; therefore, the effects of the four GTPBP3 protein mutations on cellular respiration were examined. The human tracheal epithelial cell line Beas-2B was used as a cell model. CRISPR-Cas9-mediated *GTPBP3* knockout (KO) in Beas-2B cells completely suppressed the expression of GTPBP3 (Fig. S10A).

To reveal the effect of *GTPBP3* KO on cellular respiration, oxygen consumption rate (OCR) measurements were performed in Glc- and Gal-containing media. In the Glc-containing medium, *GTPBP3* KO decreased OCR (Fig. S10B), indicating a defect in oxidative phosphorylation. Cells mainly rely on oxidative phosphorylation for energy supply when treated with Gal as the primary carbon source rather than glucose. Similarly, *GTPBP3* ablation led to reduced OCR (Fig. S10C), indicating *GTPBP3* depletion damaged cellular respiration.

To clarify the expression level of mitochondrial proteins in response to *GTPBP3* mutations, GTPBP3 plasmids harboring WT, p.R189H, p.Q262P, p.N291I, and p.E510Q mutations were transfected in *GTPBP3*-KO cell lines. Western blot was then performed to evaluate the protein levels of OXPHOS subunits, including mtDNA-encoding polypeptides (Nd5, Nd6, Cytb, and Co2), the nuclear-encoding protein Atp5c, tRNA modification and protein synthesis (Mto1 and Trmu), and mitochondrial proteins of non-OXPHOS subunits (Kars, Yars2, and Tfam). Compared with the WT, the protein levels of Nd5, Nd6, Cytb, Co2, Atp5c, Tfam, Mto1, Trmu, Kars, and Yars2 were significantly reduced when transfected with p.N291I and p.E510Q compared with transfection with p.R189H and p.Q262P (Fig. 1K–L), demonstrating that the four *GTPBP3* mutations led to deficits in mitochondrial protein expression. Meanwhile, the level of GTPBP3 was minimally altered in *GTPBP3* KO cell lines transfected with the WT, p.R189H, p.Q262P, p.N291I, and p.E510Q plasmids (Fig. 1K). Furthermore, the pathogenic model comprises heterozygous mutations; therefore, double mutations in *GTPBP3* were generated for functional studies. The protein levels of Nd5, Nd6, Cytb, and Co2 were analyzed under the WT, p.N291I, p.N291I + p.E510Q, and p.R189H + p.Q262P mutations in *GTPBP3*. The results showed that Nd5, Nd6, Cytb, and Co2 levels were significantly reduced in p.N291I + p.E510Q-expressing cells compared with those expressing the p.N291I mutation only (Fig. S11). Furthermore, the rescue assays confirmed the effects on the expression of Nd5, Atp5c, Mto1, and Kars in *GTPBP3*-KO cells upon the overexpression of these mutations (Fig. S12).

OCR measurements were conducted to further investigate the functions of the four GTPBP3 mutations. OCR was significantly reduced in cells expressing p.N291I and p.E510Q in the Glc medium. A relatively slight reduction in OCR was observed in cells expressing p.R189H and p.Q262P (Fig. 1M). In response to the Gal-containing culture medium, similar phenotypes were observed in cells expressing the four mutant plasmids (Fig. 1N). Furthermore, the effects of *GTPBP3* mutations on the enzymatic activities of respiratory complexes I and IV were also evaluated. The enzymatic activities of complexes I and IV were more prominently impaired in cells expressing p.N291I and p.E510Q, and a relatively mild inhibition was observed when cells were transfected with p.R189H and p.Q262P (Fig. 1O, P). These data demonstrated a causal role for *GTPBP3* mutations in oxidative metabolism defects, therefore, the pathogenicity of the four mutations was reclassified (Table S4).

In conclusion, we described two unrelated COXPD23 cases with severe to mild clinical phenotypes, which were probably caused by mutations in *GTPBP3*. Except for p.Q262P, the others were novel mutations. The four mutations impaired the normal protein function of GTPBP3 in mediating cell respiration and mitochondrial protein expression. These findings will offer more information on the molecular diagnosis and genetic counseling of patients with COXPD23, broaden the genetic spectrum of *GTPBP3*, and provide an accurate genetic basis for prenatal diagnosis in the future. Furthermore, whole exome sequencing may still be the first choice in clinical practice to solve complex clinical problems and provide relatively accurate genetic diagnosis. When the pathogenicity of the variant is of uncertain significance but consistent with the clinical phenotype of the patient, functional studies may be appropriately performed to reclassify the pathogenicity of the variant(s).

## Conflict of interests

The authors have no conflict of interests to declare.

## Funding

This work was supported by the 10.13039/501100001809National Natural Science Foundation of China (No. 82000321, 31900502), Henan Educational Committee Program for Science and Technology Development of Universities (China) (No. 22A310022, 24A320017), Natural Science Foundation of Henan, China (No. 212300410275), Henan Medical Science and Technology Joint Building Program (China) (No. LHGJ20190229, LHGJ20190236, and LHGJ20230283), and the Medical Science and Technology Research Project of Henan Province (No. SBGJ202103079, SBGJ202302045).
