# Supplementary material for: Mutations in GTPBP3 cause aberrant mitochondrial respiration associated with combined oxidative phosphorylation deficiency 23
Source: Genes Dis. 2024 Feb 2;12(1):101232. doi: 10.1016/j.gendis.2024.101232 (PMC11471209; doi:10.1016/j.gendis.2024.101232)
Supplement: Multimedia component 1 [file mmc1.docx]

**Supplementary Information for**

**Mutations in GTPBP3 cause aberrant mitochondrial respiration associated with combined oxidative phosphorylation deficiency 23**

Qianqian Li, Yang Yang, Ranran Li, Chenguang Yu, Kaidi Ren, Yin Feng, Xing Chen, Yi* Luan*, Xiangdong Kong*

*Correspondence: E-mail address: [kongxd@263.net](mailto:kongxd@263.net) (X.D Kong); [luan_yi@126.com](mailto:luan_yi@126.com) (Y. Luan); or [chenxing1212@163.com](mailto:chenxing1212@163.com) (X. Chen)

**This PDF file includes:**

# The clinical information of the two patients

# Materials and Methods

# Supplementary Tables 1–9

# Supplementary Figures 1–13

# The clinical information of the two patient #1 and patient #2

# Patient #1 (Ⅱ3) of family 1 was a boy who is 6 months and 18 days old. Before being hospitalized in Shanxi Maternal and Child Health Hospital on 17 June 2019, without obvious inducement, he had suffered from a poor mental state and poor eating accompanied by hyperspasmia for more than 10 days. Moreover, three days before admission, he had increased sleep and occasionally coughed and choked on milk. After hospitalization, the results of blood analysis suggested he had severe metabolic acidosis (Table S5–8 and Fig. S13), which was difficult to recover. During hospitalization, his blood pressure fluctuated around 80/25 mmHg, and he exhibited symptoms of liver failure (Table S9), hypokalemia (blood potassium = 3.31 mmol/L), myocardial injury (Table S9), and moderate anemia (hemoglobin concentration = 82 g/L). He was discharged from the hospital on 21 June 2019 and eventually died on 30 June 2019.

# The elder sister (Ⅱ1) died 11 years ago without clear genetic reasons. However, they exhibited both similar and different clinical symptoms. The similarities were: 1) their mother’s self-reported gestational week was less than the average gestational week, and 2) their global developments were slow and retarded. The differences were: 1) after birth, the elder sister had difficulty during feeding, could not raise her head on her own at the age of 3 months (which may be due to hypotonia), and her gross motor skills did not develop at all; 2) the boy could raise his head and turn over on his own; however, for unknown reasons, he was sleepy, with a poor spirit, and had difficulty during feeding.

# Patient #2 (Ⅱ1) of family 2 was a boy born on 18 December 2020. When the mother was 3 months pregnant, the nuchal translucency (NT) measurement failed twice. During her 8^th^ month of pregnancy, an ultrasound showed that the fetus’s age was only 6‒7 months. The mother had a Caesarean section in the 38^th^ week of pregnancy. After birth, the baby’s weight increased slowly.

# Patient #2 developed symptoms of dyspnea at 1.5 months old and was hospitalized at Zhoukou Central Hospital. The results of computed tomography (CT) screening suggested he had severe oropharyngeal stenosis, pectus excavatum, and pneumonia. However, he had normal muscle tone. Finally, he was transferred to Henan Children’s Hospital on 24 January 2021. The main complaint was that he had difficulty breathing for 14 days due to a respiratory tract infection. The specific manifestations included obvious chest depression accompanied by phlegm in the throat, occasional cough, choking milk, and decreased milk intake. After a series of medical examinations, he was diagnosed with severe pneumonia, (upper) cleft palate, congenital laryngeal chondromalacia, respiratory failure, atrial septal defect (ASD); continuous interruption of about 4.8 mm in the middle of the atrial septum), and suspected Pierre Robin syndrome. He was discharged on 5 February 2021. On 23 February 2021, the parents visited our center for genetic counseling, hoping to find the underlying genetic reasons.

# In addition, patient #2 underwent cleft palate repair surgery in our hospital on 19 March 2022. Two months after discharge, his weight was 12 kg and his height was 81 cm. His spirit was very good, and he responded well to the questions raised by the pediatrician He knew the location of the things he wanted and responded by shouting his name. When he played with things attentively, he only responded after repeatedly shouting his name.

# Materials and methods

# Ethics statement

# This study was approved by the ethics committee of the First Affiliated Hospital of Zhengzhou University (Ethics No. 2021-KY-0291-002). Written informed consent was obtained from the minor(s)’ legal guardian/next of kin to publish any potentially identifiable images or data included in this study.

# Whole exome sequencing (WES) and bioinformatics analysis

DNA libraries were constructed using Illumina library construction and capture kits (Illumina, San Diego, California, USA), following the manufacturer’s instructions. The quality and purity of DNA libraries were assessed using Qubit 4.0 (Thermo Fisher Scientific Inc., Massachusetts, USA) and Bio-Fragment Analyzer Qsep100 (Bioptic, Taiwan, China) systems. Then, 150-bp paired-end sequencing was conducted on a NovaSeq 6000 platform (Illumina) with the S1 flowcell for 300 cycles. Mapping, variant calling, and variant annotation were conducted using the Efficient Genosome Interpretation System (EGIS, Sierra Vast Bio-Medical, Shanghai, China, Fig. S1). After variant filtering, candidate mutations in *GTPBP3* (NM_133644.4) were identified in the two individuals, which were then verified via Sanger sequencing using the primer pairs in Supplementary Table 4. The protein structure of GTPBP3 was downloaded from the Protein Data Bank (PDB) database (https://www.rcsb.org/). PyMOL (https://pymol.org/2/) was then used for the structure prediction map of the four mutations.

# Construction of *GTPBP3* knockout (KO) cell lines and plasmids

A *GTPBP3*-KO cell line was constructed using the CRISPR/Cas9 system. Sense and antisense oligonucleotides encoding *GTPBP3* sgRNA (S: 5ʹ-CACCGACCATCTTCGCGCTAAGCTC-3ʹ, A: 5ʹ-AAACGAGCTTAGCGCGAAGATGGT-3ʹ) were cloned into the *p*X330 vector (Addgene plasmid 42230). The human tracheal epithelial cell line Beas-2B (American Type Culture Collection, Manassas, Virginia, USA) was transfected with 300 ng of *p*X330 containing *GTPBP3* sgRNA and 100 ng of modified pLL3.7 containing a puromycin resistance gene. After transfection for 24 hours, cells were treated with 1 μg mL^−1^ puromycin. After 14 days of puromycin selection, individual cell colonies were picked and transferred for further culture.

The full-length coding region of *GTPBP3* (NM_133644.4) was amplified and sub-cloned into the pEGFP-C1 vector, named pEGFP-C1-GTPBP3-WT. Four mutant plasmids were constructed using PCR-based site-directed mutagenesis with the primer pairs in Supplementary Table 8, and named pEGFP-C1-GTPBP3-p.R189H, pEGFP-C1-GTPBP3-p.Q262P, pEGFP-C1-GTPBP3-p.N291I, and pEGFP-C1-GTPBP3-p.E510Q.

**Western blot**

Cell extracts were derived from the homogenate after treatment in lysis buffer (150 mM NaCl, 1% NP-40, 0.5% sodium deoxycholate, 0.1% SDS, and 50 mM Tris-HCl, pH 7.0) containing protease inhibitors and 1 mM phenylmethanesulfonyl fluoride. Proteins were resolved via SDS-PAGE and transferred to a polyvinyl difluoride (PVDF) membrane, followed by blocking in 5% bovine serum albumin in phosphate-buffered saline containing 0.1% Triton X-100. For immunodetection, the following primary antibodies were used: anti-GTPBP3 (1:1000, HPA042158, Sigma, St. Louis, USA), anti-Nd5 (1:1000, ab92624, Abcam, Cambridge, UK), anti-Nd6 (1:500, ab81212 Abcam), anti-Atp5c (1:500, 60284-1-Ig, Proteintech, Chicago, USA), anti-Tfam (1:1000, 19998-1-AP, Proteintech), anti-Mto1 (1:500, 15650-1-AP, Proteintech), anti-Trmu (1:500, HPA000824, Sigma), anti-Cytb (1:1000, A9762, ABclonal, Wuhan, China), anti-Co2 (1:1000, 55070-1-AP, Proteintech), anti-Kars (1:1000, 14951-1-AP, Proteintech), anti-Yars2 (1:1000, ab228957, Abcam), and anti-β-actin (1:3000, A2066, Sigma). After conjugating with the corresponding secondary antibodies goat anti-mouse IgG (Sigma) and goat anti-rabbit IgG (Sigma), protein signals were analyzed using an ECL system (Thermo Fisher Scientific Inc.). Relative protein levels were evaluated using ImageJ software.

## Oxygen consumption rate (OCR) measurements

## OCR was detected on an XF24 Extracellular Flux Analyzer (Seahorse Bioscience, Billerica, MA) following the manufacturer’s guidelines. Briefly, cells were pre-adapted in a culture medium containing glucose and plated at a density of 40,000 cells/well for 24 hours. The cells were then cultured in a Glc XF24 assay (containing DMEM supplemented with 25 mM glucose, 31 mM NaCl, 1 mM sodium pyruvate, 2 mM GlutaMAX [Gibco], and 15 mg/L phenol red) and Gal XF24 assay medium (containing all the components of the Glc XF24 medium but with 10 mM galactose instead of glucose). OCR measurements were performed for 90 minutes after replacement with fresh XF24 medium. Inhibitors of the electron transport chain (ETC) and OXPHOS were also administered: oligomycin A (1 μM), CCCP (1.5 μM), rotenone (0.5 μM), and antimycin A (0.5 μM). After OCR measurements, the protein content in cells was measured using a Bio-Rad Bradford assay kit (P0006, Beyotime, Beijing, China).

## Complex I and IV enzyme activity measurements

The enzyme activity of complexes I (Complex I Enzyme Activity Microplate Assay Kit, #ab109721, Abcam) and IV (Complex IV Enzyme Activity Microplate Assay Kit, #ab109909, Abcam) was measured according to the respective manufacturer’s instructions. Enzyme activity is shown as the change in absorbance per minute (OD/minute) per 200 μg of cell lysate.

## Statistical analysis

Data are presented as the mean ± SEM. Statistical analysis was performed using GraphPad Prism 6 software. One-way ANOVA and two-tailed Student’s t-tests were used for multiple-group comparisons and between-group comparisons, respectively. Differences were statistically significant at *P* < 0.05 (**P* < 0.05, ***P* < 0.01, ****P <* 0.001).

**Supplementary Table 1.** Genetic and clinical information of individuals with *GTPBP3* mutations (NM_133644.4) (hg19/GRCh37).

| Mutation type | Genome position (chr19:) | DNA change | Protein change | Patient ID | Sex/  onset age | Presenting symptoms | Plasma  lactate (mmol/L) | TTE | Brain MRI | Outcomes, follow-up  time, cause of death | Ref |
| --- | --- | --- | --- | --- | --- | --- | --- | --- | --- | --- | --- |
| Missens/  Nonsense | 17448428 | c.8G>T | p.R3L | #82790 | F/1 y | Developmental delay, epileptic seizures, hypotonia | 5.7-6.5 | Normal | Bilateral hyperintensities in thalamus | Alive, 2 y | [1] |
|  | 17448950 | c.187C>T | p.R63* | #00305941 | - | - | - | - | - | - | [2] |
|  | 17449372 | c.413C>T | p.A138V | #00305941 | - | - | - | - | - | - | [2] |
|  |  |  |  | Patient #1 | M/17 h | Hypothermia, poor response, respiratory failure, cardiogenic shock, metabolic acidosis | 26 | Normal | - | Died, 5 d,  CHF | [3] |
|  | 17449383 | c.424G>A | p.E142K | #81471 | M/4 w | Hypothermia, recurrent apnea metabolic acidosis | 11 | HCM | Hyperintensities in subthalamic nuclei | Died, 5 w, acidosis | [1] |
|  |  |  |  | Patient #3 | F/1 y | Developmental delay, intellectual disability, fatigability | 4.26-16 | HCM | Bilateral lesions in brain stem, thalamus and cerebellum | Alive, 3 y | [3] |
|  | 17449435 | c.476A>T | p.E159V | #66143 | M/2 y | Sudden respiratory failure, CHF | - | HCM | - | Alive, 5 y | [1] |
|  | 17449443 | c.484G>C | p.A162P | #72425 | F/3.5 m | Poor feeding, failure to thrive, hypoactivity | 23.3 | DCM | - | Died, 8 m, CHF | [1] |
|  | 17449503 | c.544G>T | p.G182* | Patient #2 | F/1 y | Developmental delay, hypotonia | 7.7-14 | - | Bilateral lesions in brain stem, thalamus and cerebellum | Alive, 3 y | [3] |
|  | 17449525 | c.566G>A | p.R189H | Patient #2 | M/1.5 m | Intrauterine growth retardation, severe oropharyngeal stenosis, pectus excavatum, pneumonia | 3.2 | ASD | Normal | Alive, 1.5 y | This study |
|  | 17449940 | c.769G>A | p.E257K | #72425 | F/3.5 m | Poor feeding, failure to thrive, hypoactivity | 23.3 | DCM | - | Died, 8 m, CHF | [1] |
|  | 17449956 | c.785A>g | p.Q262P | Patient #1 | F/6 m | Poor spirit, poor feeding, increased sleep, hyperspasmia | 7.94-12.61 | - | - | Died, 7 m,  multiple organ failure | This study |
|  |  |  |  | Patient #2 | F/1 y | Developmental delay, hypotonia | 7.7-14 | - | Bilateral lesions in brain stem, thalamus and cerebellum | Alive, 3 y | [3] |
|  |  |  |  | Patient #3 | F/1 y | Developmental delay, intellectual disability, fatigability | 4.26-16 | HCM | Bilateral lesions in brain stem, thalamus and cerebellum | Alive, 3 y | [3] |
|  | 17450037 | c.866C>A | p.P289H | #75168 | F/2 y | Developmental delay, epileptic seizures | >10 | - | Bilateral hyperintensities in thalamus | Alive, 5 y | [1] |
|  | 17450043 | c.872A>T | p.Q291I | Patient #1 | F/6 m | Poor spirit, poor feeding, increased sleep, hyperspasmia | 7.94-12.61 | - | - | Died, 7 m,  multiple organ failure | This study |
|  | 17450270 | c.932C>T | p.P311L | Patient No. 24 | F/3 w | Mental motor retardation, seizure, hearing disability, thrombocytopenia | - | Normal | Delayed myelination | Alive, 10 y | [4] |
|  | 17450398 | c.1060G>C | p.A354P | #72425 | F/3.5 m | Poor feeding, failure to thrive, hypoactivity | 23.3 | DCM | - | Died, 8 m, CHF | [1] |
|  |  |  |  | #66143 | M/2 y | Sudden respiratory failure, CHF | - | HCM | - | Alive, 5 y |  |
|  | 17451887 | c.1105G>C | p.D369H | #75191 | F/birth | Poor feeding, hypotonic,  respiratory failure | 23 | HCM | - | Died, 1 d, asystolia | [1] |
|  | 17452408 | c.1471G>A | p.E491K | #36349 | M/- | Intellectual disability, fatigability, visual impairment, slight dyspnea with climbing stairs | - | HCM | Lactate peaks in parietal and precentral cortex | Alive, 17 y | [1] |
|  |  |  |  | #49665 | M/10 y | Intellectual disability, fatigability, visual impairment, slight dyspnea with climbing stairs | 3-7 | HCM | Lactate peaks in parietal and precentral cortex | Alive, 14 y |  |
|  | 17452465 | c.1528G>C | p.E510Q | Patient #2 | M/1.5 m | Intrauterine growth retardation, severe oropharyngeal stenosis, pectus excavatum, pneumonia | 3.2 | ASD | Normal | Alive, 1.5 y | This study |
|  | 17452472 | c.1535T>A | p.I512N | Patient #80 | - | Global developmental delay, hypotonia, microcephaly, nystagmus, and elevated lactic acid | - | - | - | - | [5] |
| Splicing | 17449930 | c.761-2delA | p.A222G,  p.D223_S270del; p.A222G;  p.D223_S270del | #76671 | M/birth | Poor feeding, hypotonic, CHF, metabolic acidosis | 5.2 | HCM | Bilateral hyperintensities in thalamus | Died, 10 m, CHF | [1] |
| Small insertions | 17450271_17450272 | c.933_934dupGG | p.E312Gfs*23 | #4287-1 | F/- | Schizophrenia | - | - | - | - | [6] |
|  | 17452324 | c.1387dupC | p.R463Pfs*87 | #36349 | M/- | Intellectual disability, fatigability, visual impairment, slight dyspnea with climbing stairs | - | HCM | Lactate peaks in parietal and precentral cortex | Alive, 17 y | [1] |
|  |  |  |  | #49665 | M/10 y | Intellectual disability, fatigability, visual impairment, slight dyspnea with climbing stairs | 3-7 | HCM | Lactate peaks in parietal and precentral cortex | Alive, 14 y |  |
| Small indels | 17448452_17448453 | c.32_33delAAinsGTG | p.Q11Rfs*98 | #83904 | F/1 w | Cardiogenic shock, metabolic acidosis | 20 | DCM | - | Died, 9 m, CHF | [1] |
|  |  |  |  | #83905 | F/birth | Cardiogenic shock, metabolic acidosis | - | DCM | - | Died, 10 d, CHF |  |
|  | 17449468_17449469 | c.509_510delAG | p.E170Gfs*42 | Patient #1 | M/17 h | Hypothermia, poor response, respiratory failure, cardiogenic shock, metabolic acidosis | 26 | Normal | - | Died, 5 d  CHF | [3] |
| Gross deletions | 17450357_17450381 | c.1019_1043del24 | p.E341Rfs*10 | Pt751 | F/1-6 m | Leigh disease (seizures, developmental delay) | - | - | Abnormalities of the basal ganglia | - | [7] |
|  | 17450368_17450391 | c.1030_1053del24 | p.E341_Q348del | #82790 | F/1 y | Developmental delay, epileptic seizures, hypotonia | 5.7-6.5 | Normal | Bilateral hyperintensities in thalamus | Alive, 2 y | [1] |
| Gross insertions | - | - | - | - | - | - | - | - | - | - | - |
| Complex | - | - | - | - | - | - | - | - | - | - | - |
| Repeats | - | - | - | - | - | - | - | - | - | - | - |
| Regulatory | - | - | - | - | - | - | - | - | - | - | - |
| Small deletions | - | - | - | - | - | - | - | - | - | - | - |

TTE: transthoracic echocardiography; F: female; M: male; y: year; m: month; w: week; d: day; h: hour; HCM: hypertrophic cardiomyopathy; DCM: dilated cardiomyopathy; ASD: atrial septal defect; -: not available; Ref: Reference.

[1] Robert Kopajtich, Thomas J Nicholls, Joanna Rorbach et al. Mutations in GTPBP3 cause a mitochondrial translation defect associated with hypertrophic cardiomyopathy, lactic acidosis, and encephalopathy. *Am J Hum Genet*. 2014 Dec 4;95(6):708-720.

[2] Tianwen Zhu, Xiaohui Gong, Fei Bei et al. Application of next-generation sequencing for genetic diagnosis in neonatal intensive care units: results of a multicenter study in China. *Front Genet*. 2020 Nov 6;11:565078.

[3] Hui-Ming Yan, Zhi-Mei Liu, Bei Cao et al. Novel Mutations in the GTPBP3 Gene for Mitochondrial Disease and Characteristics of Related Phenotypic Spectrum: The First Three Cases From China. *Front Genet*. 2021 Jul 1;12:611226.

[4] M Elmas, H Yıldız, M Erdoğan et al. Comparison of clinical parameters with whole exome sequencing analysis results of autosomal recessive patients; a center experience. *Mol Biol Rep*. 2019 Feb;46(1):287-299.

[5] Ahmed Alfares, Majid Alfadhel, Tariq Wani et al. A multicenter clinical exome study in unselected cohorts from a consanguineous population of Saudi Arabia demonstrated a high diagnostic yield. *Mol Genet Metab*. 2017 Jun;121(2):91-95.

[6] Menachem Fromer, Andrew J Pocklington, David H Kavanagh et al. De novo mutations in schizophrenia implicate synaptic networks. *Nature*. 2014 Feb 13;506(7487):179-184.

[7] Masakazu Kohda, Yoshimi Tokuzawa, Yoshihito Kishita et al. A Comprehensive Genomic Analysis Reveals the Genetic Landscape of Mitochondrial Respiratory Chain Complex Deficiencies. *PLoS Genet*. 2016 Jan 7;12(1):e1005679.

**Supplementary Table 2.** Quality control of the whole exome sequencing (WES) data.

Family 1

| 20 × Coverage (%) | | | Data size (Mb) | | | Capture efficiency (%) | | | Duplication rate (%) | | | Average sequencing depth | | |
| --- | --- | --- | --- | --- | --- | --- | --- | --- | --- | --- | --- | --- | --- | --- |
| Ⅱ3 | Ⅰ1 | Ⅰ2 | Ⅱ3 | Ⅰ1 | Ⅰ2 | Ⅱ3 | Ⅰ1 | Ⅰ2 | Ⅱ3 | Ⅰ1 | Ⅰ2 | Ⅱ3 | Ⅰ1 | Ⅰ2 |
| 97.40 | 97.15 | 98.05 | 8703 | 8428 | 8862 | 76.71 | 77.08 | 80.55 | 0.199 | 0.187 | 0.196 | 106.18 | 103.67 | 115.09 |

Family 2

| 20 × Coverage (%) | Data size (Mb) | Capture efficiency (%) | Duplication rate (%) | Average sequencing depth |
| --- | --- | --- | --- | --- |
| 99.1 | 12661 | 76.86% | 0.149 | 152.22 |

**Supplementary Table 3.** Primer sequences used for Sanger sequencing and the construction of mutant plasmids.

| Primer name | Primer sequence (Forward) | Primer sequence (Reverse) |
| --- | --- | --- |
| *GTPBP3*-c.566G>A-Sequencing | 5′- GGCTGTGCTGTCCTCCTGTC-3′ | 5′- GCGGGATTGAGAGGGGGT-3′ |
| *GTPBP3*-c.785A>C-Sequencing | 5′-CCCCCATTTGTCCATTCTCTCCC-3′ | 5′-GGGGGAGGGTCAGAAGAAGAGGAG-3′ |
| *GTPBP3*-c.872A>T-Sequencing |  |  |
| *GTPBP3*-c.1528G>C-Sequencing | 5′-ACCCGAGCAAGGCACCAG-3′ | 5′-TCCCAGGCGAATGAGAATCC-3′ |
| *GTPBP3*-c.566G>A-MU | 5′-TGGGCCACCTCTGCCATGGCTGGGCCGAGAC-3′ | 5′-GTCTCGGCCCAGCCATGGCAGAGGTGGCCCA-3′ |
| *GTPBP3*-c.785A>C-MU | 5′-AAGTACGGGCACTGCCGGTGGCCCTGGGTGC-3′ | 5′-GCACCCAGGGCCACCGGCAGTGCCCGTACTT-3′ |
| *GTPBP3*-c.872A>T-MU | 5′-TCACTGGACCCCCCATTGCGGGCAAGAGCAG-3′ | 5′-CTGCTCTTGCCCGCAATGGGGGGTCCAGTGA-3′ |
| *GTPBP3*-c.1528G>C-MU | 5′-GGTGGAGGGGGTACCCAGGAGATCCTGGACA-3′ | 5′-TGTCCAGGATCTCCTGGGTACCCCCTCCACC-3′ |

**Supplementary Table 4.** The pathogenicity of the four mutations according to the latest guidelines of the ACMG (before and after the functional studies).

| Patient ID | Variant information | Evidence of pathogenicity (before) | ACMG classification (before) | Evidence of pathogenicity (after) | ACMG classification (after) |
| --- | --- | --- | --- | --- | --- |
| Patient #1 | c.785A>C(p.Q262P) | PS4_Supporting+PM1+PM2_Supporting | VUS | PS4_Supporting+PS3_Supporting+PM1+PM2_Supporting | P |
|  | c.872A>T(p.N291I) | PM1+PM2_Supporting+PP3 | VUS | PS3_Supporting+PM1+PM2_Supporting+PP3 | LP |
| Patient #2 | c.566G>A(p.R189H) | PM1+PM2_Supporting+BP4 | VUS | PS3_Supporting+PM1+PM2_Supporting+BP4 | LP |
|  | c.1528G>C(p.E510Q) | PM2_Supporting+PM1 | VUS | PS3_Supporting+PM2_Supporting+PM1 | LP |

VUS: variant of uncertain significance; P: pathogenic; LP: likely pathogenic.

**Supplementary Table 5.** Blood analysis of patient #1.

| Project | 18 June (00:01) | 18 June (22:05) | 19 June (07:25) | 19 June (14:13) | 19 June (17:30) | 20 June (09:16) | 21 June (09:14) | Units | Reference |
| --- | --- | --- | --- | --- | --- | --- | --- | --- | --- |
| pH | 7.160 ↓ | 7.150 ↓ | 7.090 ↓ | 7.170 ↓ | 7.32 ↓ | 7.400 | 7.390 | - | 7.350-7.450 |
| pCO_2_ | 14.0 ↓ | 11.0 ↓ | 18.0 ↓ | 21.0 ↓ | 22.0 ↓ | 22.0 ↓ | 24.0 ↓ | mmHg | 35.0-45.0 |
| pO_2_ | 115.0 ↑ | 129.0 ↑ | 113.0 ↑ | 125.0 ↑ | 141.0 ↑ | 164.0 ↑ | 194.0 ↑ | mmHg | 80.0-100.0 |
| BE | -21.5 ↓ | -22.5 ↓ | -22.7 ↓ | -19.3 | -13.5 ↓ | -10.1 ↓ | -9.7 ↓ | mmol/L | -2-+3 |
| TCO_2_ | 5.5 ↓ | 4.2 ↓ | 5.8 ↓ | 8.1 ↓ | 11.6 ↓ | 14.1 ↓ | 14.9 ↓ | mmol/L | 22-29 |
| HCO3^-^ | 5.0 ↓ | 3.9 ↓ | 5.2 ↓ | 7.4 ↓ | 11.0 ↓ | 13.4 ↓ | 14.2 ↓ | mmol/L | 18-23 |
| BB | 24.8 ↓ | 24.2 ↓ | 22.3 ↓ | 26.3 ↓ | 32.0 ↓ | 35.0 ↓ | 35.3 ↓ | mmol/L | 46-52 |
| BE(act) | -21.8 ↓ | -22.8 ↓ | -22.3 ↓ | -19.6 ↓ | -13.3 ↓ | -9.6 ↓ | -9.2 ↓ | mmol/L | -2-+3 |
| BE(ecf) | -23.7 ↓ | -24.9 ↓ | -24.6 ↓ | -21.6 ↓ | -15.1 ↓ | -11.4 ↓ | -10.8 ↓ | mmol/L | -2-+3 |
| stHCCO3 | 8.0 ↓ | 7.1 ↓ | 7.5 ↓ | 9.7 ↓ | 13.7 ↓ | 16.2 ↓ | 16.6 ↓ | mmol/L | 19-24 |
| st.PH | 6.923 ↓ | 6.870 ↓ | 6.898 ↓ | 7.008 ↓ | 7.158 ↓ | 7.232 ↓ | 7.242 ↓ | - | 7.350-7.450 |
| cH^+^ | 69.1 ↑ | 70.0 ↑ | 81.6 ↑ | 68.0 ↑ | 47.8 ↑ | 39.8 | 41.0 | mmol/L | 36-44 |
| Na^+^ | 136.0 | 134.0 ↓ | 135.0 | 136.0 | 139.0 | 144.0 | 141.0 | mmol/L | 135.0-145.0 |
| K^+^ | 3.5 | 4.4 | 3.3 ↓ | 3.3 ↓ | 4.1 | 3.4 ↓ | 4.1 | mmol/L | 3.50-5.10 |
| nCa^2+^ | 1.06 ↓ | 1.16 | 0.99 ↓ | 1.01 ↓ | 1.02 ↓ | 1.22 | 1.21 | mmol/L | 1.12-1.27 |
| tHb | 15.0 | 15.0 | 15.0 | 15.0 | 15.0 | 15.0 | 15.0 | g/dL | 12.0-17.0 |
| SO2 | 97.0 | 98.0 | 96.0 | 98.0 | 99.0 | 99.0 | 99.0 | % | 90-100 |
| Hct | 33 ↓ | 35 | 31 ↓ | 29 ↓ | 27 ↓ | 24 ↓ | 23 ↓ | % | 34-51 |
| AaDO2 | 2.7 ↓ | 0.0 ↓ | 0.2 ↓ | 0.0 ↓ | 0.0 ↓ | 0.0 ↓ | 0.0 ↓ | mmHg | 5-20 |
| O_2_Ct | 14.9 ↓ | 16.4 | 14.0 ↓ | 13.3 ↓ | 12.9 ↓ | 11.7 ↓ | 11.4 ↓ | Vol% | 15-23 |
| P50(C) | 34.9 ↑ | 34.0 ↑ | - | 33.8 ↑ | 30.8 ↑ | 32.5 ↑ | - | Vol% | 25-29 |

**Supplementary Table 6.** Lactic acid (LAC), pyruvic acid (PYR), blood ammonia (AMM), aninon gap (AG), and hydroxybutyric dehydrogenase (HBDH) levels in the blood of patient #1.

| LAC | Reference  (mmol/L) | PYR | Reference  (μmol/L) | AMM | Reference  (μmol/L)  90 | AG | Reference | HBDH | Reference  (U/L) |
| --- | --- | --- | --- | --- | --- | --- | --- | --- | --- |
| 12.61 (17 June) ↑ | 0.6-2.2 | 565.8 (17 June) ↑ | 20-100 | 46.9 (17 June) | 10-47 | - | 8-16 | - | 72-182 |
| 12.47 (19 June) ↑ |  | 95.4 (19 June) |  | 29.6 (19 June) |  | 20 (19 June) ↑ |  | 1850 (19 June) ↑ |  |
| 7.94 (20 June) ↑ |  | 20 (20 June) |  | 17.2 (19 June) |  | 13 (19 June) |  | 2290 (19 June) ↑ |  |

**Supplementary Table 7.** Biochemical test results of the cerebrospinal fluid of patient #1 on 20 June 2019.

| Project |  | Unit | Reference |
| --- | --- | --- | --- |
| Glucose (GLU) | 5.68 ↑ | mmol/L | 2.8-4.5 |
| Chlorine (CL) | 128.4 | mmol/L | 120-132 |
| Protein | 0.38 | g/L | 0.15-0.45 |
| Lactic acid (LC) | 10.55 ↑ | mmol/L | 1.1-2.8 |
| Lactic dehydrogenase (LDH) | 455 ↑ | U/L | <50 |

**Supplementary Table 8.** Comprehensive analysis of organic acid levels in the urine of patient #1 on 17 June 2019.

| Project |  | Reference (μM) |  | Project |  | Reference (μM) |  |
| --- | --- | --- | --- | --- | --- | --- | --- |
| Lactic acid-2 | 152.7 | 0.0-13.0 | ↑ | 2-hydroxyisobutyrc acid-2 | 0.0 | 0.0-5.0 |  |
| Caproic acid-1 | 0.0 | 0.0-1.0 |  | Glycolic acid | 5.8 | 0.0-8.0 |  |
| Oxalic acid-2 | 8.3 | 0.0-33.0 |  | 2-hydroxybutyric acid-2 | 69.1 | 0.0-2.0 | ↑ |
| Glyoxylic acid-OX-2 | 4.2 | 0.0-65.0 |  | 3-hydroxypropionic acid-2 | 9.9 | 0.0-4.0 | ↑ |
| Pyruvic acid-OX-2 | 242.5 | 0.0-30.0 | ↑ | Valproic acid-1 | 0.0 | 0.0-4.0 |  |
| 3-hydroxybutyric acid-2 | 451.1 | 0.0-9.0 | ↑ | 3-hydroxyisobutyric acid-2 | 15.0 | 0.0-9.0 | ↑ |
| 2-hydroxyisovaleric acid-2 | 41.7 | 0.0-2.0 | ↑ | 2-methyl-3-hydroxybutyric acid-2 | 0.0 | 0.0-4.0 |  |
| Malonic acid-2 | 0.0 | 0.0-0.0 |  | 3-hydroxyisovaleric acid-2 | 4.5 | 0.0-4.0 | ↑ |
| 2-ketoisovaleric acid-OX-2 | 11.2 | 0.0-0.3 | ↑ | Methyl malonic acid-2 | 0.0 | 0.0-4.0 |  |
| 2-ethyl-3-hydroxypropionic acid-2 | 29.3 | 0.0-6.0 | ↑ | Urea-2 | 123.8 | 0.0-700.0 |  |
| 4-hydroxybutyric acid-2 | 0.0 | 0.0-1.0 |  | 2-hydroxy isosolic acid-2 | 0.0 | 0.0-0.3 |  |
| 3-hydroxyvaleric acid-2 | 0.0 | 0.0-6.0 |  | Acetoacetic acid | 0.0 | 0.0-0.7 |  |
| 2-hydroxy-3-methyl pentanoic acid-2 | 0.0 | 0.0-0.2 |  | Benzoin acid-1 | 0.0 | 0.0-70.0 |  |
| Acetoacetic acid-2 | 0.0 | 0.0-1.5 |  | Caprylic acid-1 | 0.0 | 0.0-0.2 |  |
| 2-keto-3-methylvaleric acid-2 | 11.7 | 0.0-1.0 | ↑ | 2-methyl-3-hydroxyvaleric acid-2 (1) | 0.0 | 0.0-1.5 |  |
| Glycerol | 0.0 | 0.0-20.0 |  | Phosphoric acid-3 | 41.0 | 0.0-200.0 |  |
| 2-methyl-3-hydroxyvaleric acid-2 (2) | 0.0 | 0.0-1.5 |  | Ethyl malonic acid-2 | 3.4 | 0.0-7.0 |  |
| 2-ketoisohexanoic acid-OX-2 | 27.5 | 0.0-0.8 | ↑ | Acetylglycine-1 (1) | 0.0 | 0.0-0.0 |  |
| Phenylacetic acid-1 | 0.0 | 0.0-5.0 |  | Maleic acid-2 | 0.0 | 0.0-1.3 |  |
| Succinic acid-2 | 9.4 | 0.0-85.0 |  | Methyl succinic acid-2 | 0.0 | 0.0-4.0 |  |
| Glyceric acid-3 | 1.3 | 0.0-10.0 |  | Uracil-2 | 0.0 | 0.0-8.0 |  |
| Fumaric acid-2 | 43.2 | 0.0-16.0 | ↑ | Propionyl glycine-1 | 0.0 | 0.0-0.4 |  |
| Acetylglycine-1 (2) | 0.0 | 0.0-0.0 |  | Mevalonate lactone-2 | 0.0 | 0.0-0.0 |  |
| Mevalonate lactone-1 | 0.0 | 0.0-4.0 |  | Isobutyryl glycine-1 | 0.0 | 0.0-0.5 |  |
| 2-propyl-3-hydroxyvaleric acid-2 | 0.0 | 0.0-1.0 |  | Methyl fumaric acid-2 | 1.9 | 0.0-2.5 |  |
| Glutaric acid-2 | 0.0 | 0.0-8.0 |  | 3-methyl pentenedioic acid-2 | 0.0 | 0.0-1.0 |  |
| 3-methyl glutaric acid-2 | 0.0 | 0.0-3.0 |  | 2-propyl-3-keto-valeric acid-2 | 0.0 | 0.0-0.0 |  |
| Propionyl glycine-2 | 0.0 | 0.0-0.0 |  | Isobutyryl glycine-2 | 0.0 | 0.0-0.0 |  |
| 2-deoxy-4-hydroxyacetylacetic acid | 0.0 | 0.0-5.0 |  | Butyryl glycine-1 | 0.0 | 0.0-4.0 |  |
| 3-methyl pentenedioic acid-2 (1) | 8.3 | 0.0-4.0 | ↑ | Pentenedioic acid-2 | 0.0 | 0.0-0.0 |  |
| Succinyl acetone-OX-2 (1) | 0.0 | 0.0-0.0 |  | Decanoic acid-2 | 0.0 | 0.0-1.0 |  |
| 2-propyl-5-hydroxyvaleric acid-2 | 0.0 | 0.0-10.0 |  | 3-methyl pentenedioic acid-2 (2) | 3.2 | 0.0-5.0 |  |
| Isovaleryl glycine-1 | 0.0 | 0.0-1.5 |  | Butyryl glycine-2 | 0.0 | 0.0-0.5 |  |
| Malic acid-3 | 2.0 | 0.0-1.5 | ↑ | Adipic acid-2 | 4.4 | 0.0-15.0 |  |
| Isovaleryl glycine-2 | 0.0 | 0.0-0.5 |  | 2-hexenedioic acid-2 | 0.0 | 0.0-30.0 |  |
| 5-oxyproline-2 | 5.1 | 0.0-10.0 |  | 3-methyl adipic acid | 0.0 | 0.0-6.0 |  |
| Thiodiacetic acid-2 | 0.0 | 0.0-40.0 |  | 2-propyl-hydroxyglutaric acid-2 | 0.0 | 0.0-15.0 |  |
| 7-hydroxyoctanoic acid-2 | 0.0 | 0.0-4.0 |  | 5-hydroxy-methyl-2-furoic acid-1 | 2.8 | 0.0-60.0 |  |
| Methyl crotonyl glycine-2 | 0.0 | 0.0-0.5 |  | 3-methyl crotonyl glycine-1 | 0.0 | 0.0-5.0 |  |
| Methyl crotonyl glycine-1 | 0.0 | 0.0-5.0 |  | 3-methyl crotonyl glycine-2 | 0.0 | 0.0-0.5 |  |
| 2-hydroxyglutaric acid-3 | 1.4 | 0.0-4.0 |  | 3-hydroxyglutaric acid-3 | 4.3 | 0.0-15.0 |  |
| Phenyllactic acid-2 | 0.0 | 0.0-4.0 |  | Pimelic acid-2 | 2.7 | 0.0-15.0 |  |
| 3-hydroxy-3-methyl glutaric acid-3 | 0.0 | 0.0-5.0 |  | 3-hydroxyphenylacetic acid-2 | 0.0 | 0.0-15.0 |  |
| 2-ketoglutaric acid-2 (1) | 72.5 | 0.0-100.0 |  | 4-hydroxy benzoin acid-2 | 0.0 | 0.0-50.0 |  |
| 4-hydroxyphenylacetic acid | 6.4 | 0.0-140.0 |  | 2-ketoglutaric acid-2 (2) | 2.7 | 0.0-10.0 |  |
| Hexyl glycine-1 | 0.0 | 0.0-1.0 |  | Phenylpyruvic acid-OX-2 | 0.0 | 0.0-0.2 |  |
| N-acetylaspartic acid-2 | 0.0 | 0.0-3.0 |  | 2-hydroxyadipic acid-3 | 1.0 | 0.0-15.0 |  |
| Octene diacid-2 | 1.6 | 0.0-5.0 |  | 3-hydroxyadipic acid-3 | 0.0 | 0.0-3.0 |  |
| Octanedioic acid-2 | 1.4 | 0.0-10.0 |  | 3-methyl pentenedioic acid-2 (3) | 0.0 | 0.0-0.1 |  |
| 2-ketoadipic acid-OX-3 | 0.8 | 0.0-5.0 |  | Aconitic acid-3 | 72.0 | 0.0-135.0 |  |
| Whey acid-3 | 0.0 | 0.0-2.0 |  | Vanillic acid-2 | 0.0 | 0.0-70.0 |  |
| Homovanillic acid-2 | 12.1 | 0.0-80.0 |  | Azelaic acid-2 | 0.5 | 0.0-18.0 |  |
| Hippuric acid-2 | 1.7 | 0.0-70.0 |  | Isocitrate-4 | 11.7 | 0.0-20.0 |  |
| Citric acid-4 | 36.8 | 0.0-310.0 |  | Urinary melanic acid-3 | 0.0 | 0.0-6.5 |  |
| Hippuric acid-1 | 2.1 | 0.0-250.0 |  | Methyl citric acid-4 (1) | 0.0 | 0.0-0.7 |  |
| 3- (3-hydroxyphenyl)-3-hydroxypropionic acid-3 | 0.0 | 0.0-90.0 |  | Methyl citric acid-4 (2) | 0.0 | 0.0-0.8 |  |
| 3-hydroxyoctene diacid-3 | 0.0 | 0.0-5.0 |  | 3-hydroxyoctanedioic acid-3 | 5.4 | 0.0-8.0 |  |
| Urinary vanilla mandelic acid-3 | 25.6 | 0.0-100.0 |  | Sebacic acid-2 | 0.0 | 0.0-17.0 |  |
| Sundienoic acid-2 | 2.8 | 0.0-10.0 |  | 4-hydroxyphenyllactic acid-2 | 44.5 | 0.0-20.0 | ↑ |
| 4-hydroxyphenylpyruvic acid-OX-2 | 5.0 | 0.0-5.0 |  | 2-hydroxyhippuric acid-3 | 0.0 | 0.0-5.0 |  |
| Indole-3-acetic acid-2 | 0.0 | 0.0-35.0 |  | Succinyl glycine-2 | 0.0 | 0.0-0.8 |  |
| Palmitic acid-1 | 21.8 | 0.0-60.0 |  | 2-hydroxy solanedioic acid-3 | 1.8 | 0.0-12.0 |  |
| 3-hydroxysolanedioic acid-3 | 15.5 | 0.0-12.0 | ↑ | 2-hydroxyhippuric acid-2 | 0.0 | 0.0-100.0 |  |
| Dodecanedioic acid-2 | 0.0 | 0.0-0.3 |  | N-acetyltyrosine-2 | 0.0 | 0.0-6.0 |  |
| Uric acid-4 | 3.2 | 0.0-20.0 |  | 3,6-epoxydodecylic acid-2 | 0.0 | 0.0-12.0 |  |
| 3-hydroxy dodecanedioic acid-3 | 0.0 | 0.0-12.0 |  | 3,6-epoxy tetradecanoic acid-2 | 0.0 | 0.0-8.0 |  |

**Supplementary Table 9.** Biochemical detection of the peripheral blood of patient #1 on 17, 19, and 20 June 2019.

| Project | 17 June | 19 June | 20 June | Units | Reference |
| --- | --- | --- | --- | --- | --- |
| Alanine aminotransferase (ALT) | 23 | 1196 ↑ | 1505 ↑ | U/L | 9-50 |
| Aspartate aminotransferase (AST) | 50 ↑ | 2544 ↑ | 2257 ↑ | U/L | 15-40 |
| γ-glutamyltransferase (GGT) | 23 | 25 | 24 | U/L | 10-60 |
| Total protein (Tpr) | 61.3 ↓ | 42.9 ↓ | 42.1 ↓ | g/L | 65-85 |
| Albumin (Alb) | 44.2 ↓ | 31.9 ↓ | 30.2 ↓ | g/L | 40-55 |
| Globulin (GLB) | 17.10 ↓ | 11.00 ↓ | 11.90 ↓ | g/L | 20-40 |
| Alb/GLB | 2.58 ↑ | 2.90 ↑ | 2.54 ↑ |  | 1.0-2.4 |
| Total bilirubin (TBIL) | 18.6 ↑ | 19.9 ↑ | 19.3 ↑ | μmol/L | 3.4-17.1 |
| Direct bilirubin (DBIL) | 3.4 | 6.4 ↑ | 6.9 ↑ | μmol/L | 0-3.4 |
| Indirect bilirubin (IBIL) | 15.20 ↑ | 13.50 ↑ | 12.40 ↑ | μmol/L | 1.7-10.2 |
| Total bile acid (TBA) | 18.2 ↑ | 16.6 ↑ | 38.2 ↑ | μmol/L | 0-10 |
| Alkaline phosphatase (ALP) | 268 | 209 | 196 | U/L | 0-500 |
| Blood urea nitrogen (BUN) | 3.1 | 1.1 ↓ | 1.4 ↓ | mmol/L | 3.1-8.0 |
| Creatinine (Cr) | 25 ↓ | 12 ↓ | 9 ↓ | μmol/L | 57-97 |
| Total CO_2_ | 13.1 ↓ | 17.5 ↓ | 16.5 ↓ | mmol/L | 22-29 |
| Uric acid (UA) | 488 ↑ | 153 ↓ | 64 ↓ | μmol/L | 208-428 |
| cystatin C (Cys-C) | 0.91 | 0.54 ↓ | 0.56 ↓ | mg/L | 0.59-1.03 |
| K | 4.99 | 3.46 | 3.97 | mmol/L | 3.5-5.3 |
| Na | 137.1 | 143.6 | 139.7 | mmol/L | 137-147 |
| Cl | 106.4 | 105.7 | 109.8 | mmol/L | 99-110 |
| Ca | 2.78 | 2.21 | 2.28 | mmol/L | 2.23-2.80 |
| Inorganic phosphorus (P) |  | 0.54 ↓ | 0.35 ↓ | mmol/L | 1.45-2.10 |
| Mg |  | 0.88 | 0.89 | mmol/L | 0.75-1.02 |
| Total cholesterol (CHO) |  | 2.36 | 2.66 | mmol/L | ≤5.2 |
| Triglyceride (TG) |  | 0.39 | 1.25 | mmol/L | ≤1.70 |
| GLucose (GLU) | 4.43 | 6.06 | 6.36 ↑ | mmol/L | 3.9-6.1 |
| Lactate dehydrogenase (LD-L) | 207 | 2862 ↑ | 2528 ↑ | U/L | 120-250 |
| High density lipoprotein cholesterol (HDLD) |  | 0.95 ↓ | 0.95 ↓ | mmol/L | 1.16-1.42 |
| Low density lipoprotein cholesterol (LDLD) |  | 0.99 ↓ | 1.05 ↓ | mmol/L | 2.7-3.1 |
| Creatine kinase (CK) | 196 | 10750 ↑ | 6640 ↑ | U/L | 50-310 |
| Activity of creatine kinase MB isoenzyme (CK-MB) | 17 | 578 ↑ | 541 ↑ | U/L | 0-24 |
| Osmotic pressure (OSM) | 282 | 294 | 287 | Mosm | 273-300 |
| Anion gap (AG) | 18 ↑ | 20 ↑ | 13 | - | 8-16 |
| Amylase (AMS) |  | 104 | 22 ↓ | U/L | 32-135 |
| α- Hydroxybutyrate dehydrogenase (HBDH) | 195 ↑ | 1850 ↑ | 2290 ↑ | U/L | 72-182 |
| Prealbumin (PAB) | 215 | 127 ↓ | 87 ↓ | mg/L | 180-380 |
| Cholinesterase (CHE) | 7629 | 5173 | 4839 ↓ | U/L | 5000-12000 |
| Lipase (LPS) |  | 29 | 21 | U/L | 13-63 |
| Serum iron (SI) |  | 2.7 | 16.2 | μmol/L | 9-32.2 |
| Lactic acid (LAC) | 12.61 ↑ | 12.47 ↑ | 7.94 ↑ | mmol/L | 0.6-2.2 |
| Ammonia (AMM) | 46.9 | 29.6 | 17.2 | μmol/L | 10-47 |
| Pyruvic acid (PYR) | 565.8 ↑ | 95.4 | 20 | μmol/L | 20-100 |

**Supplementary Figure 1.** Graphical variation screening of the candidate variant filtering.

**
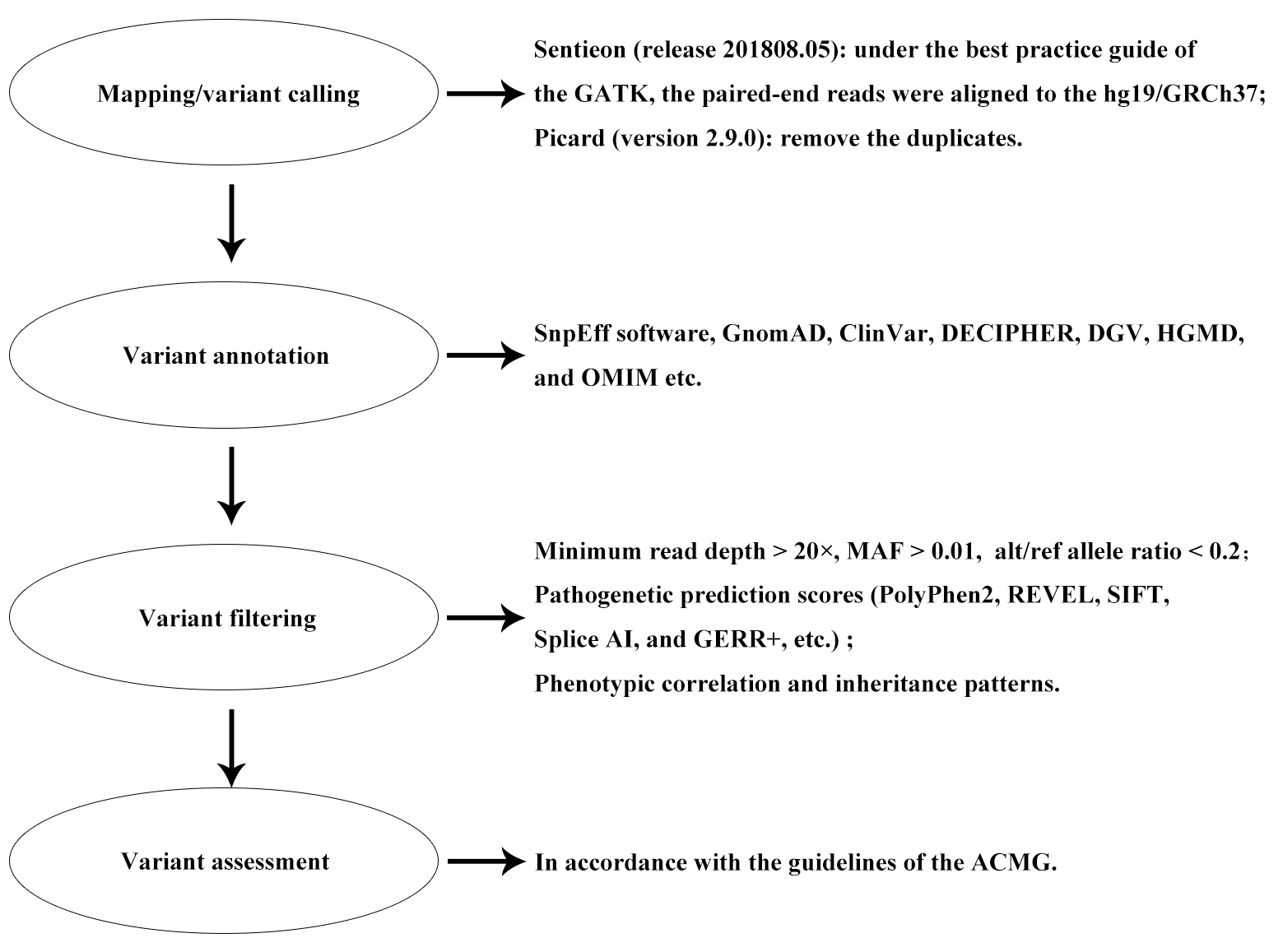
**

GATK: Genome Analysis Toolkit.

GnomAD: http://www.gnomad-sg.org/.

ClinVar: https://www.clinicalgenome.org/data-sharing/clinvar/.

DECIPHER: https://www.deciphergenomics.org/.

DGV: Database of Structural Variations in the Human Genome (http://varianttools.sourceforge.net/Annotation/DGV).

HGMD: Human Gene Mutation Database.

OMIM: Online Mendelian Inheritance in Man (https://omim.org/about).

MAF: Minor Allele Frequency.

Alt/ref allele ratio: alternative/reference allele ratio.

PolyPhen2: http://genetics.bwh.harvard.edu/pph2/index.shtml.

REVEL: Rare Exome Variant Ensemble Learner.

SIFT: http://provean.jcvi.org/protein_batch_submit.php?species=human.

**Supplementary Figure 2.** Screenshot of the exome alignment of p.Q262P in patient #1.


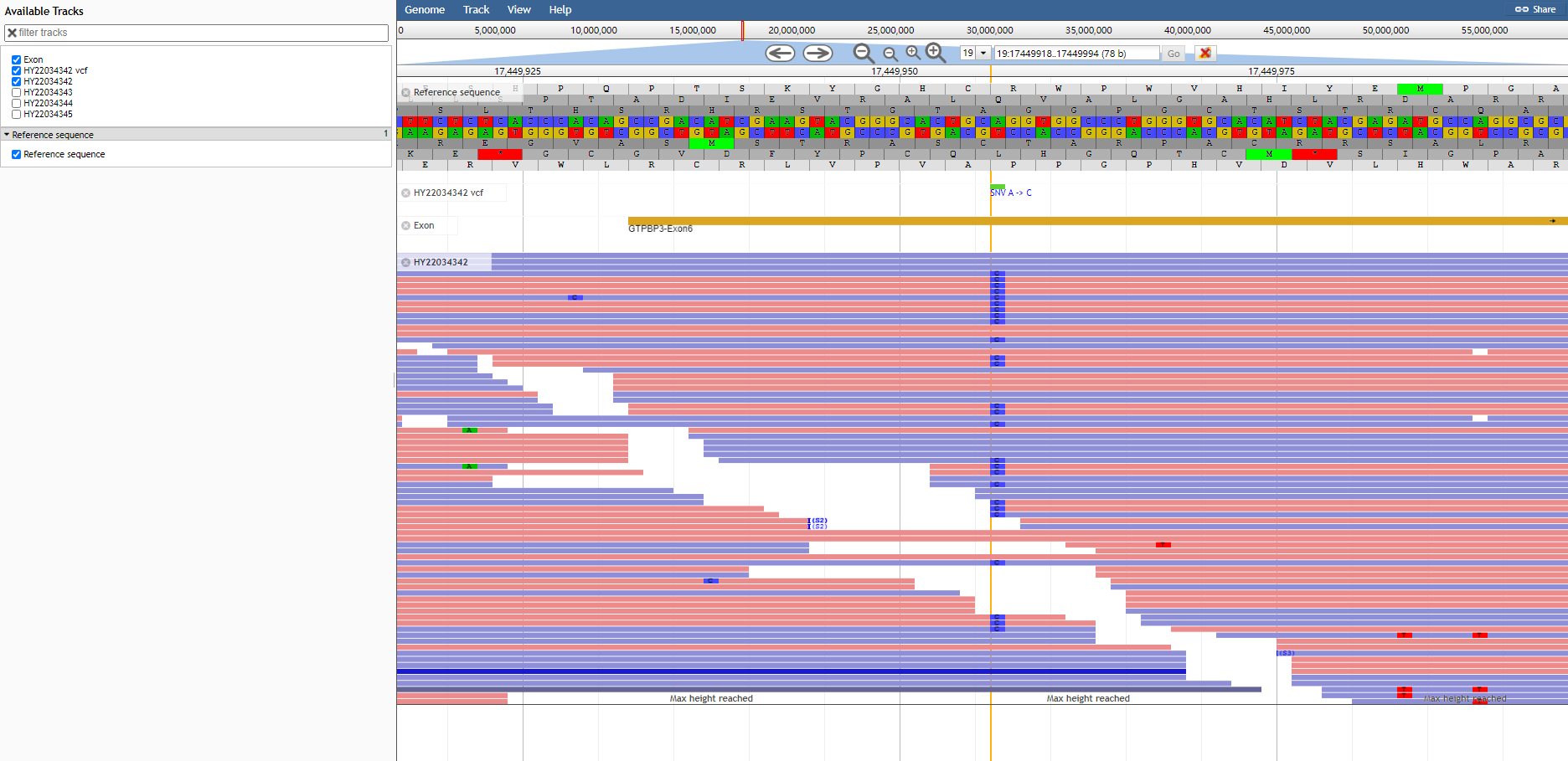


**Supplementary Figure 3.** Screenshot of the exome alignment of p.Q262P in patient #1’s mother.


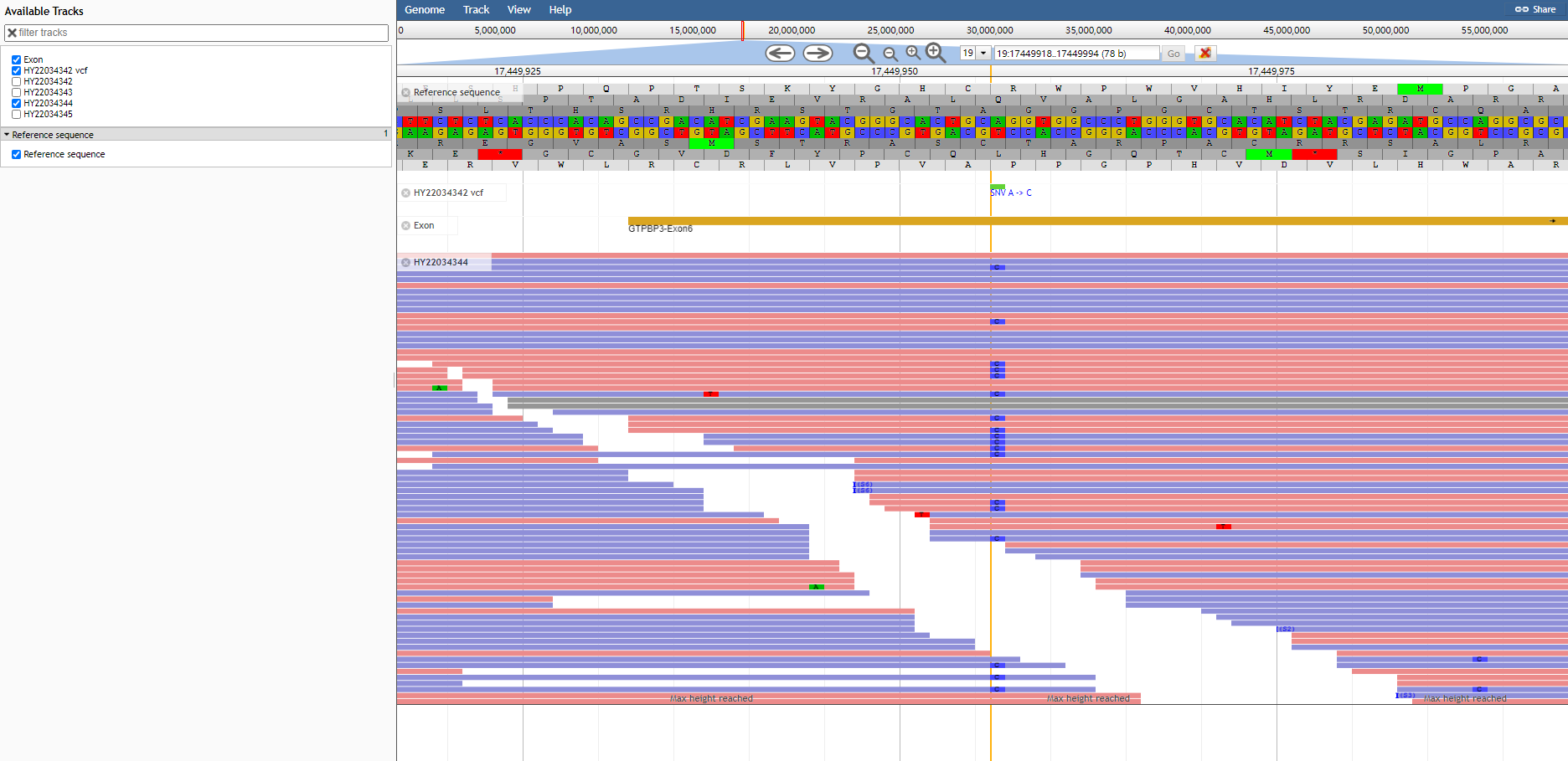


**Supplementary Figure 4.** Screenshot of the exome alignment of p.Q262P in patient #1’s father.


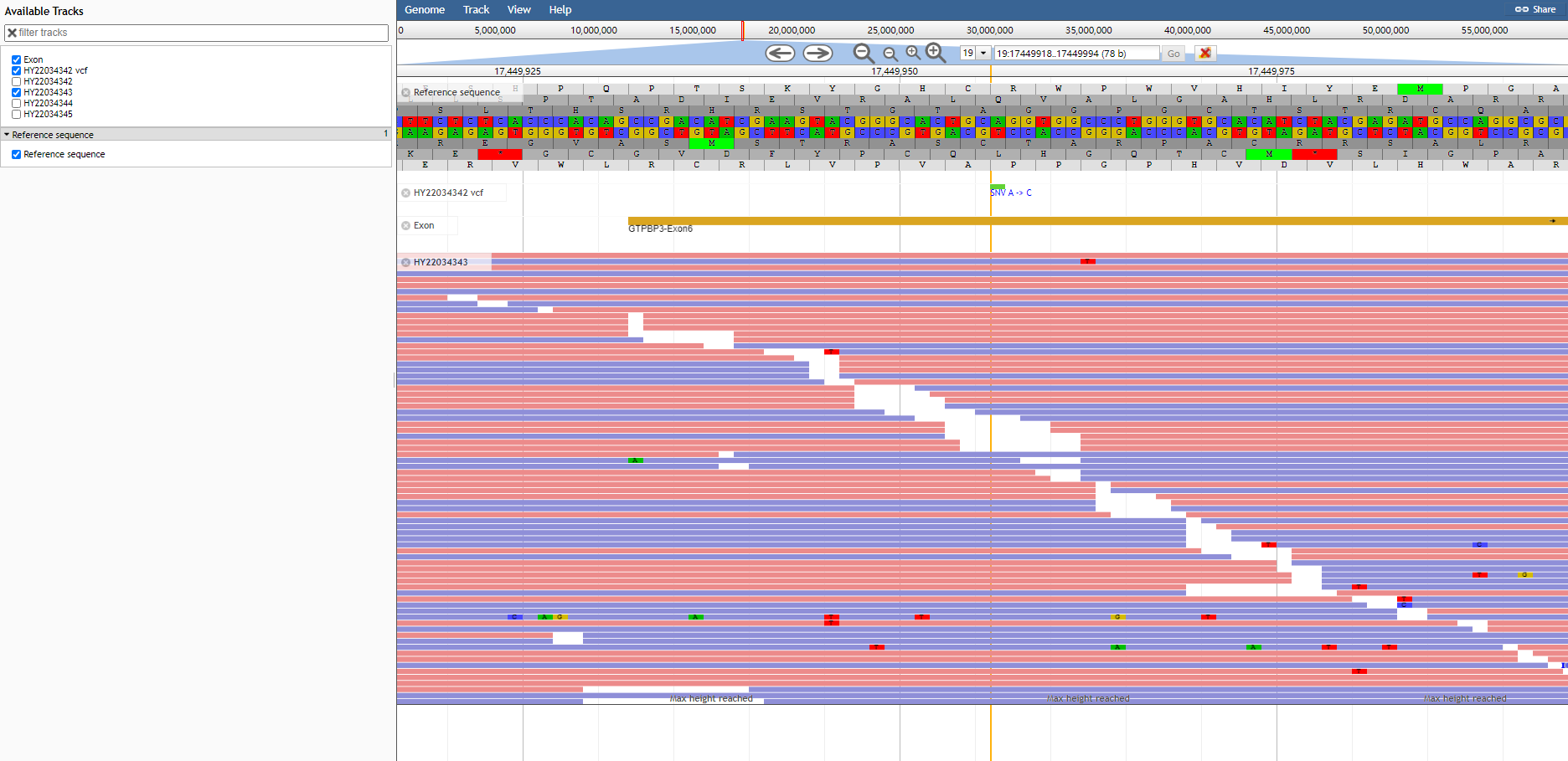


**Supplementary Figure 5**. Screenshot of the exome alignment of p.N291I in patient #1.


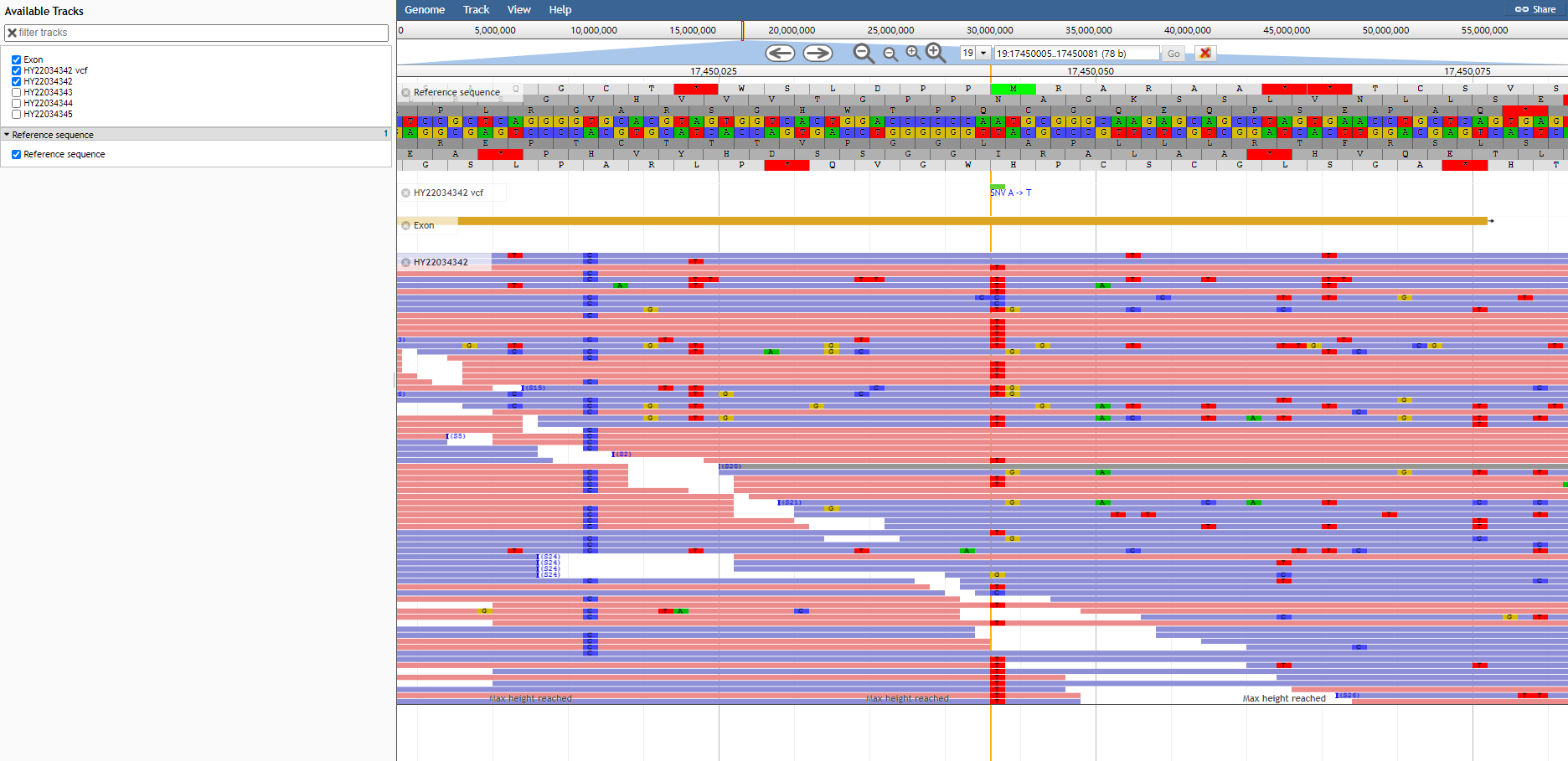


**Supplementary Figure 6.** Screenshot of the exome alignment of p.N291I in patient #1’s mother.

**
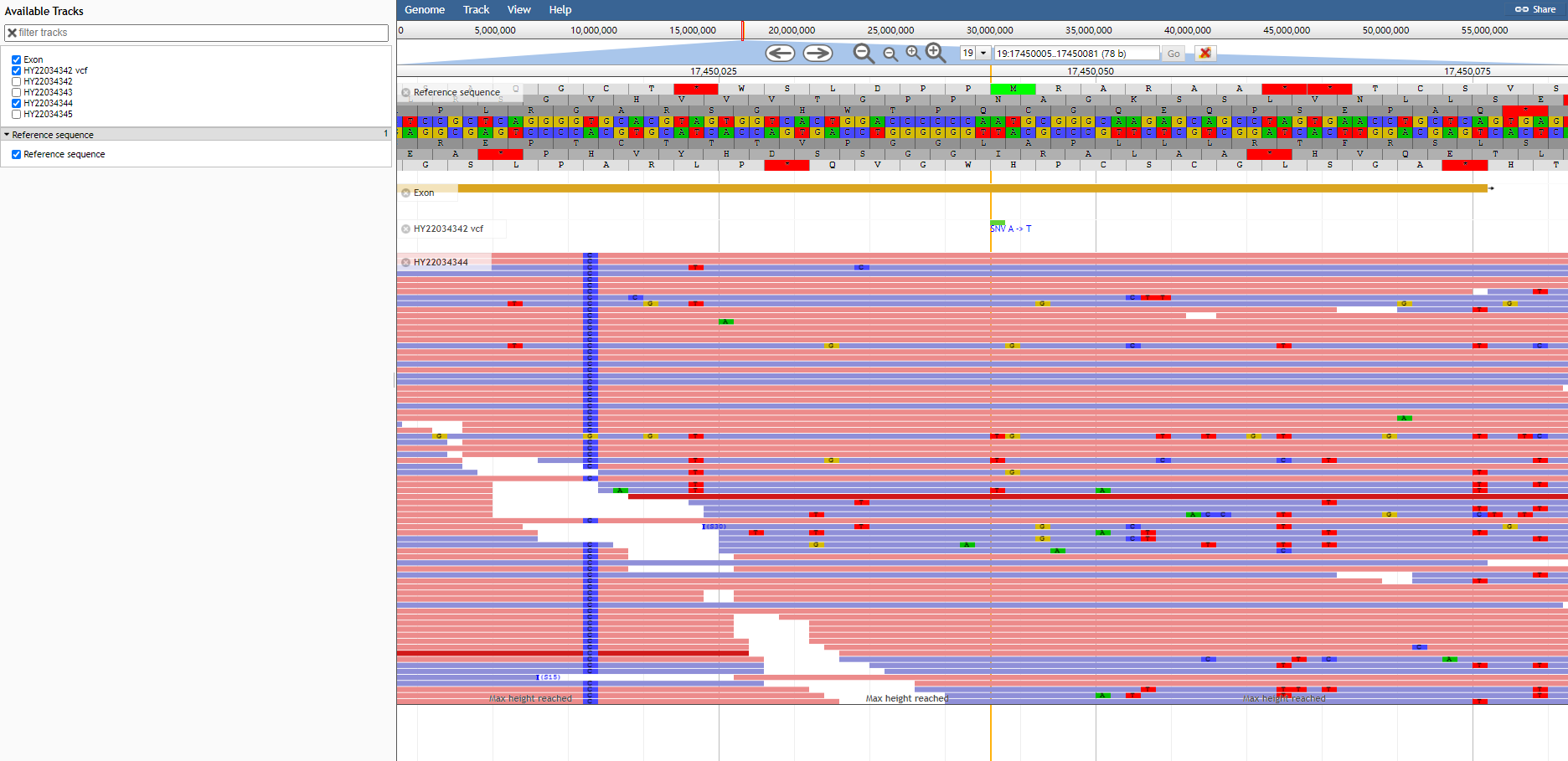
**

**Supplementary Figure 7.** Screenshot of the exome alignment of p.N291I in patient #1’s father.


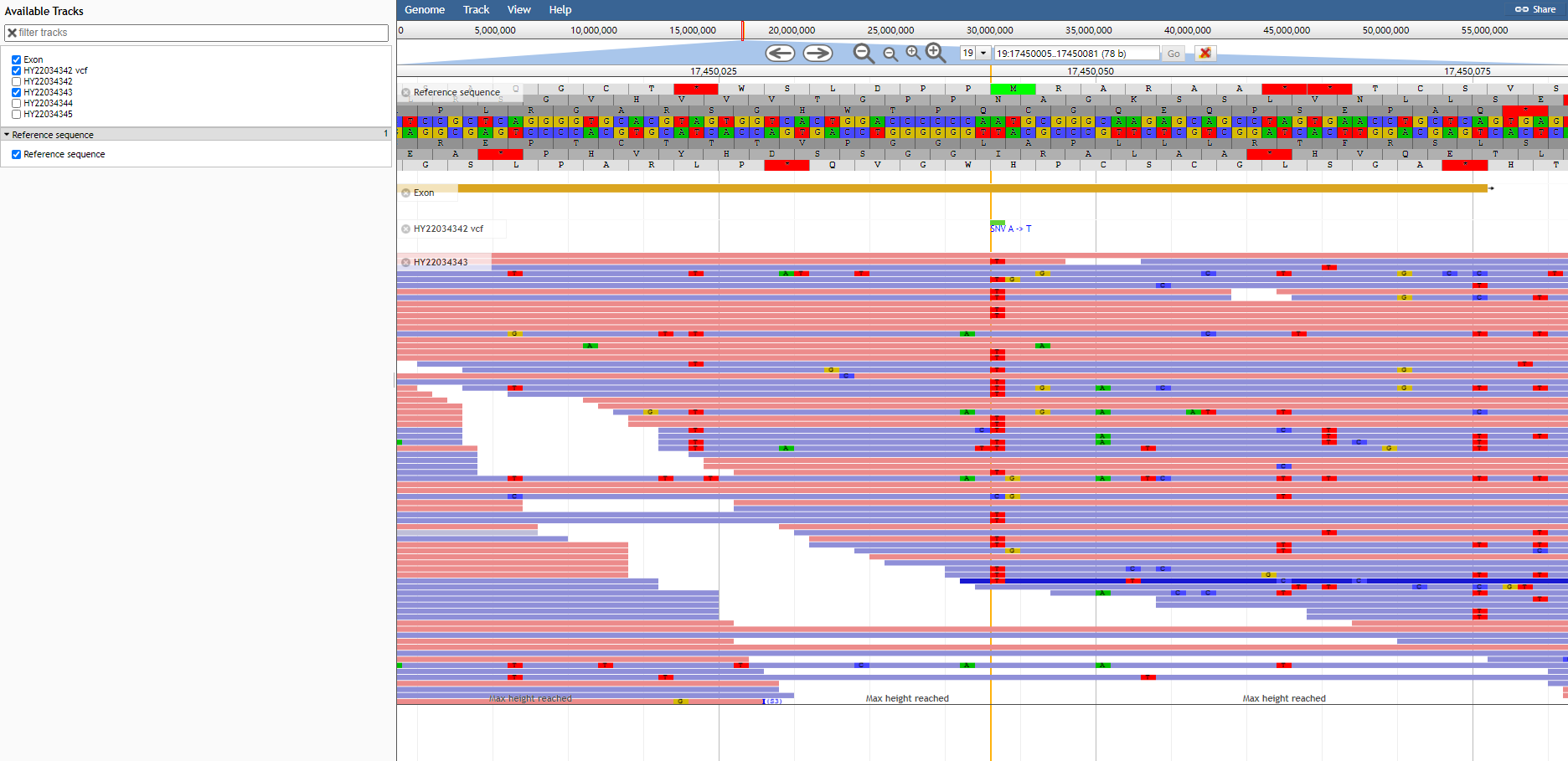


**Supplementary Figure 8.** Screenshot of the exome alignment of p.R189H in patient #2.


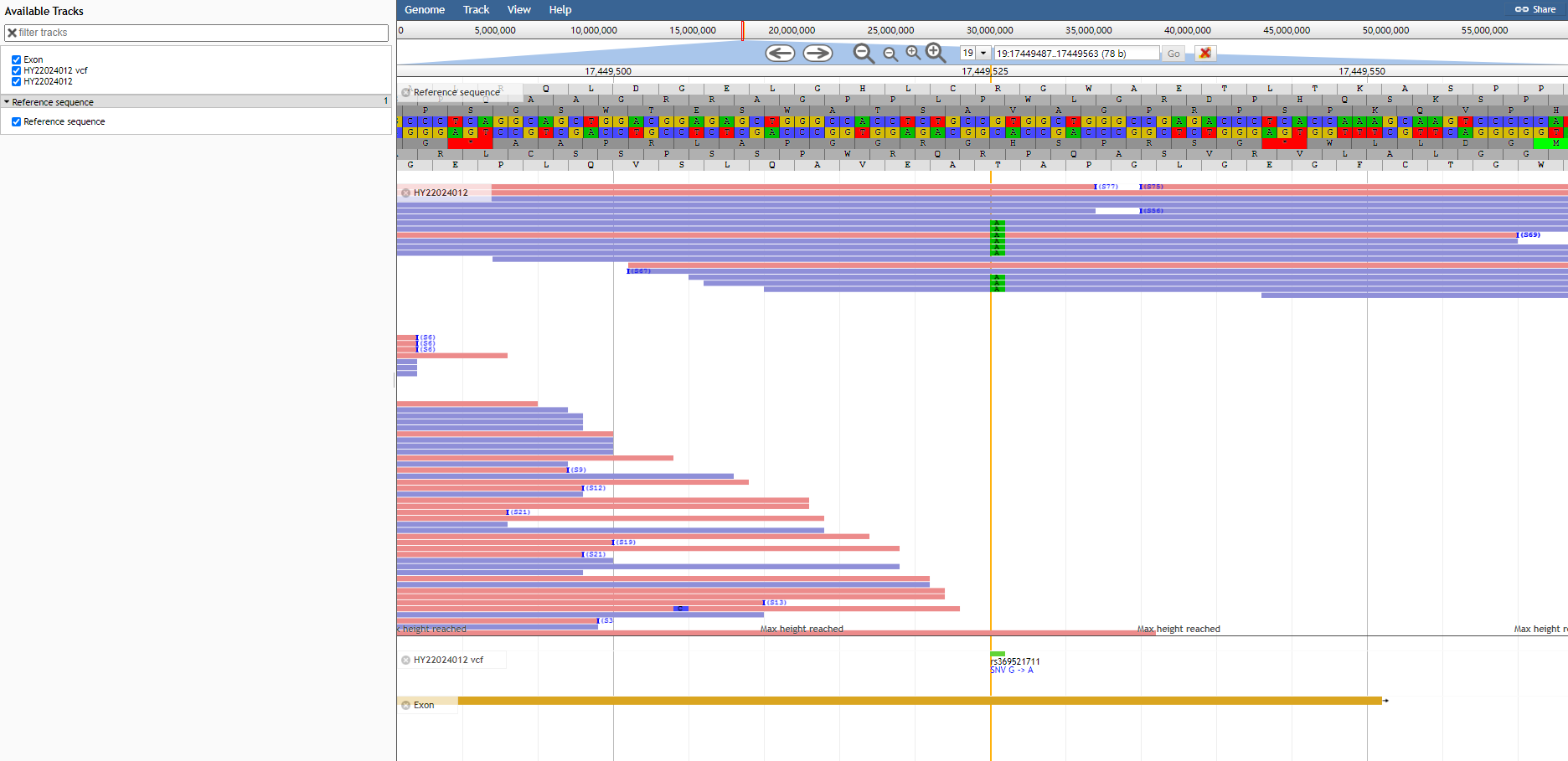


**Supplementary Figure 9.** Screenshot of the exome alignment of p.E510Q in patient #2.


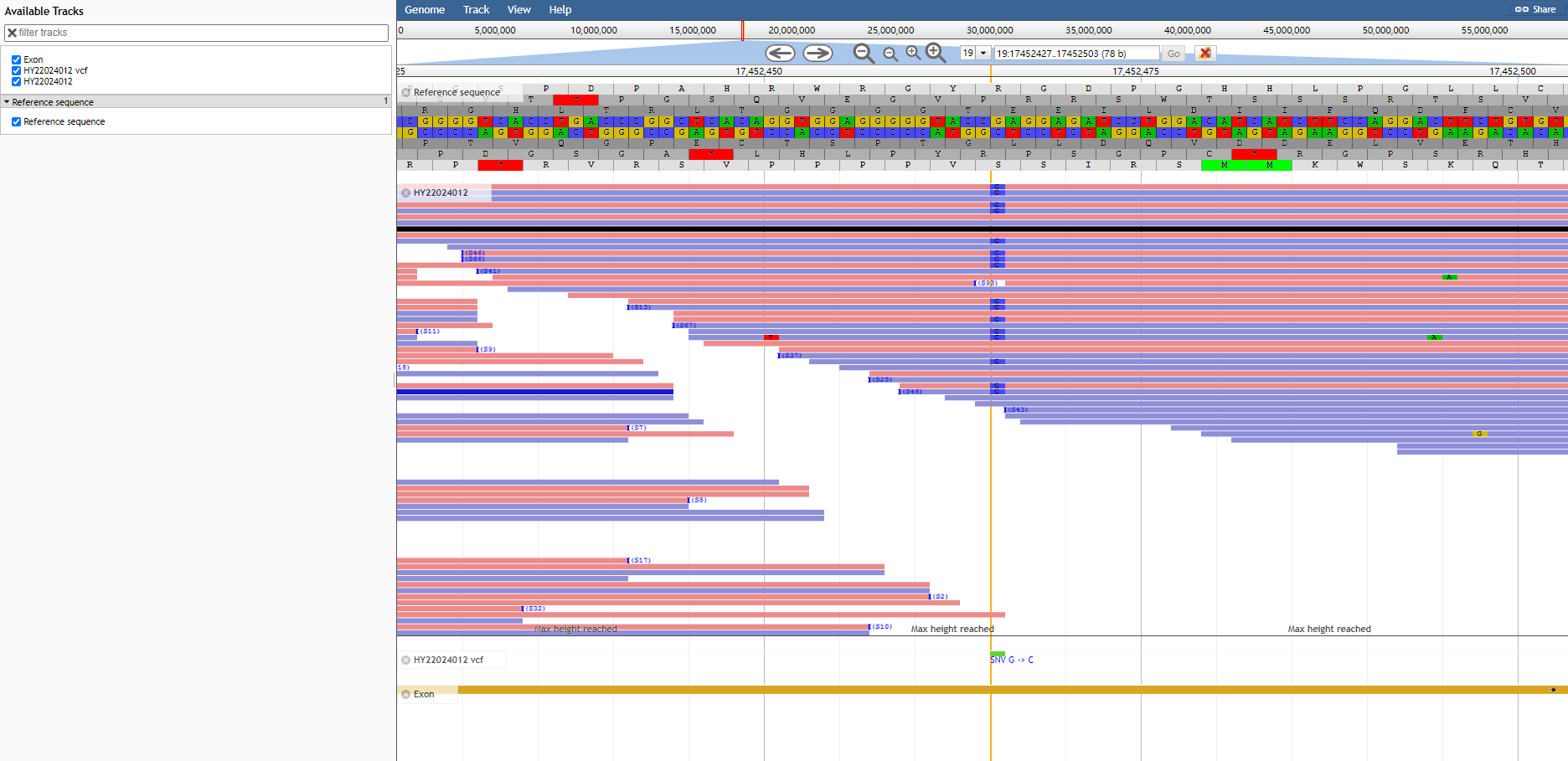


**Supplementary Figure 10**. *GTPBP3* knockout inhibited cellular respiration in lung epithelial cells.

**
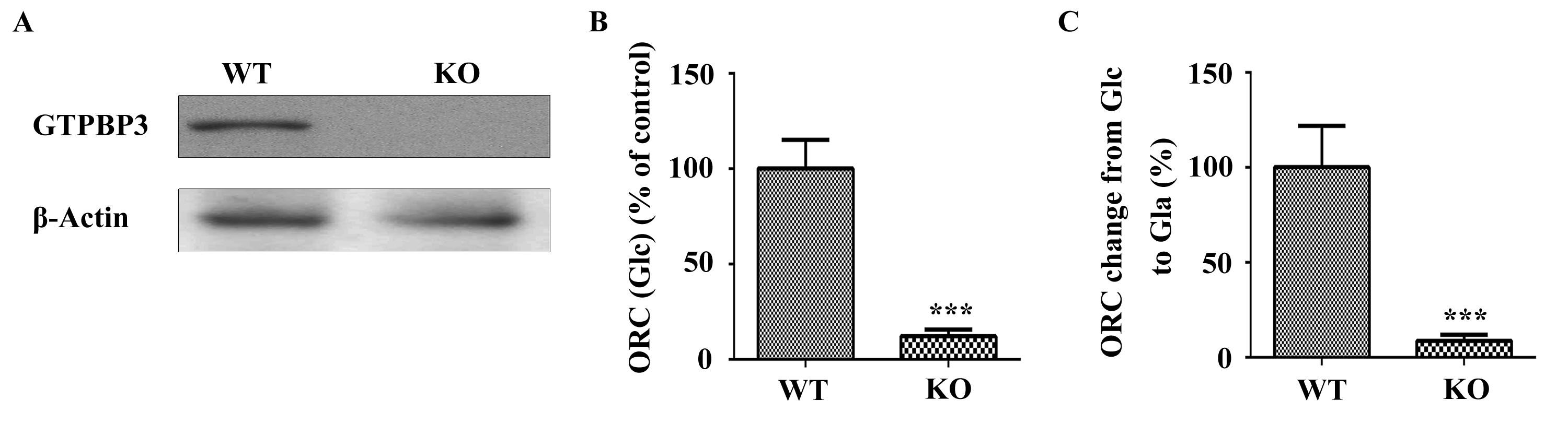
**

**(A)** *GTPBP3* was depleted in lung epithelial cells as detected via western blot. **(B)** OCR of lung epithelial cells with or without GTPBP3 expression after culture in a high-glucose (Glc) medium. OCR was expressed as a percentage relative to that of the WT. **(C)** OCR of lung epithelial cells with or without GTPBP3 expression after culture in a galactose (Gal) growth medium. ****P* < 0.001.

**Supplementary Figure 11**.The protein levels of OXPHOS in *GTPBP3*-KO cell lines treated with WT *GTPBP3* and heterozygous *GTPBP3* mutant plasmids.


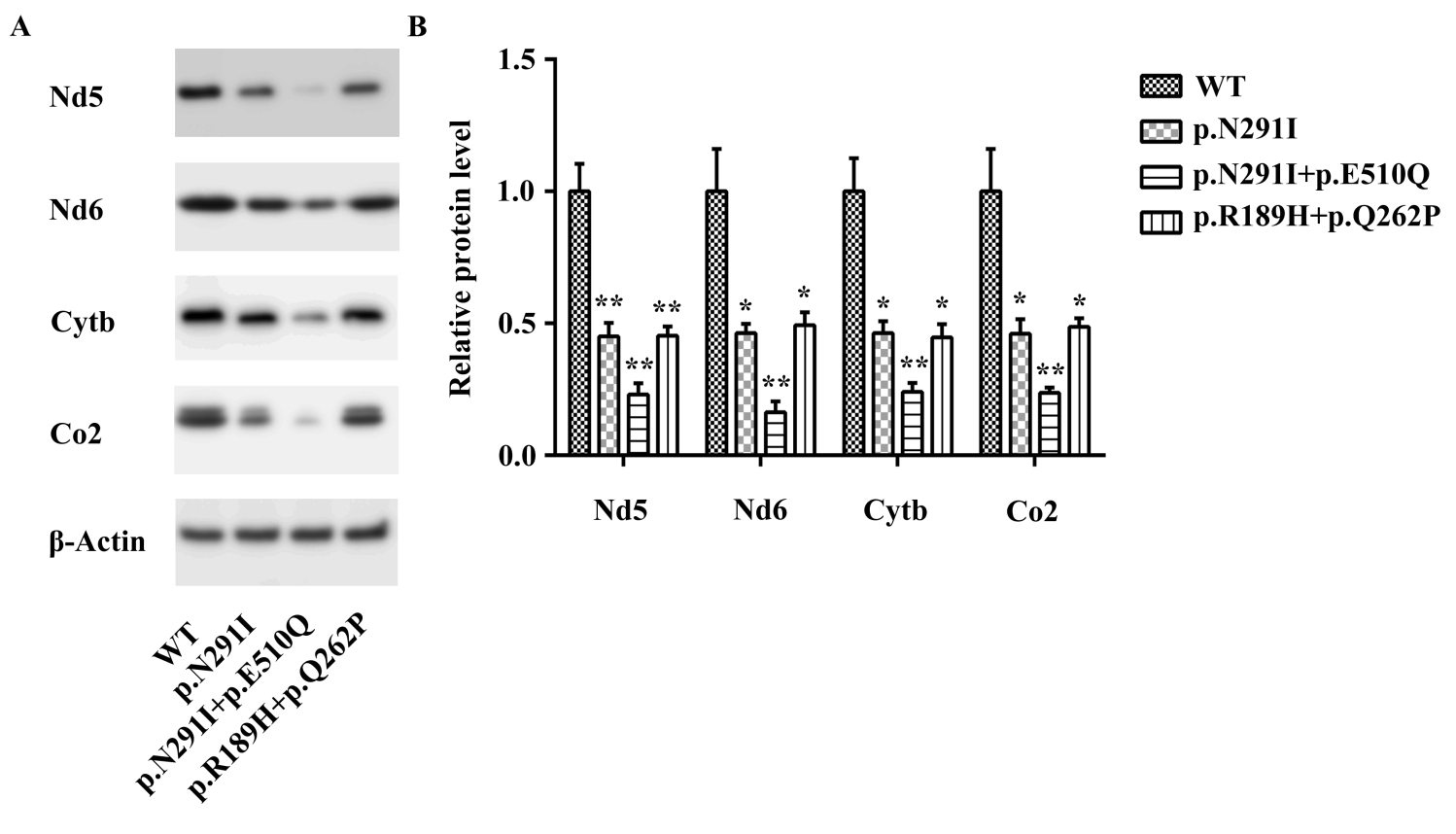


**(A, B)** Protein levels of Nd5, Nd6, Cytb, and Co2. **P* < 0.05, ***P* < 0.01.

**Supplementary Figure 12**. Effects of *GTPBP3* KO and mutations on the expression of the protein levels of OXPHOS.


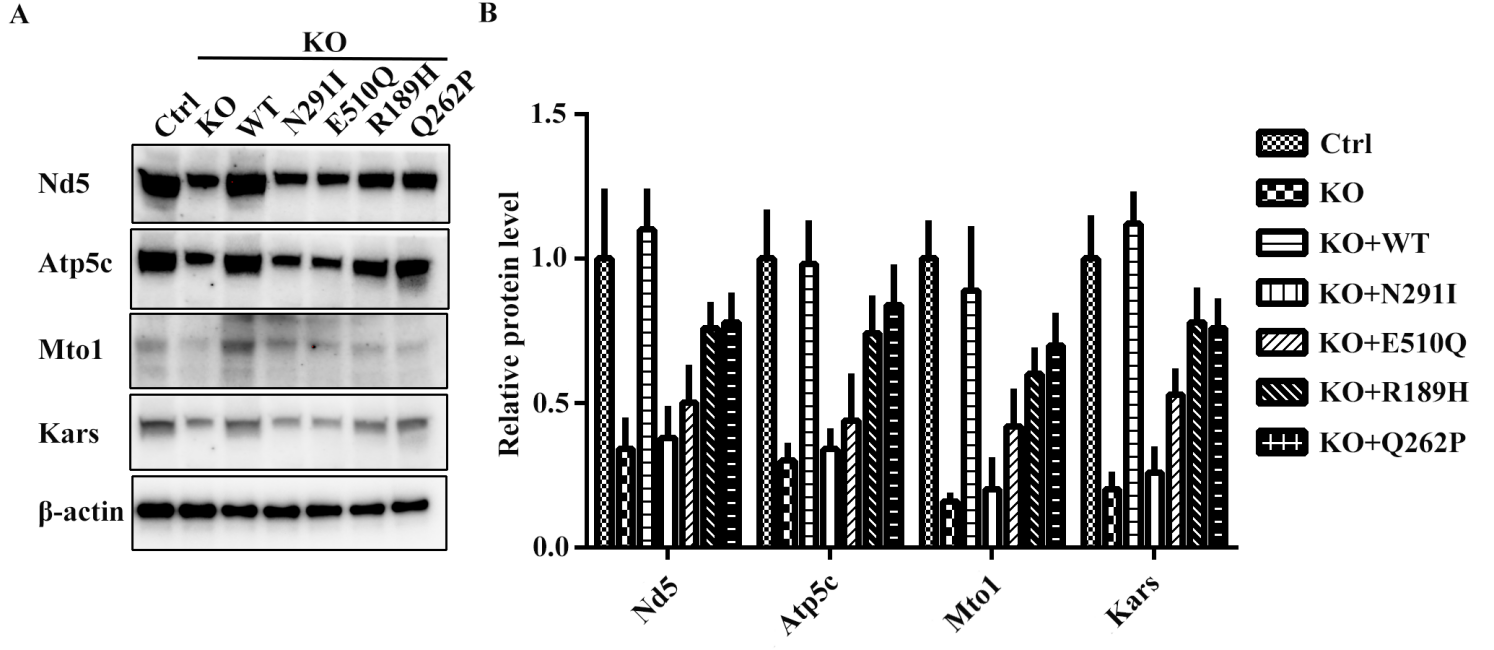


(**A, B**) Immunoblot assays showed the expression of Nd5, Atp5c, Mto1, and Kars in cells upon the indicated transfection. ***P* < 0.01.

**Supplementary Figure 13.** Comprehensive analysis of organic acid levels in the urine of patient #1 on 17 June 2019.

**
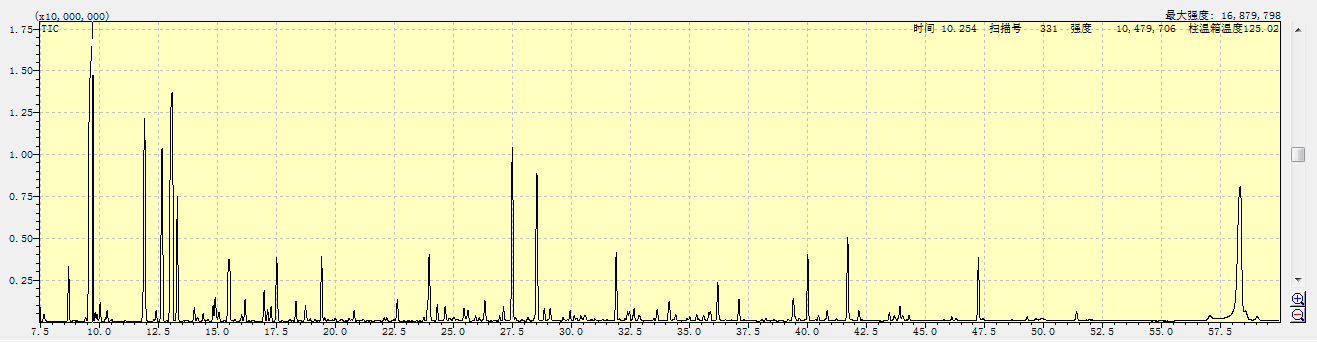
**
